# Diverse meibum lipids produced by Awat1 and Awat2 are important for stabilizing tear film and protecting the ocular surface

**DOI:** 10.1016/j.isci.2021.102478

**Published:** 2021-04-26

**Authors:** Megumi Sawai, Keisuke Watanabe, Kana Tanaka, Wataru Kinoshita, Kento Otsuka, Masatoshi Miyamoto, Takayuki Sassa, Akio Kihara

**Affiliations:** 1 Laboratory of Biochemistry, Faculty of Pharmaceutical Sciences, Hokkaido University, Sapporo, Kita 12-jo, Nishi 6-chome, Kita-ku 060-0812, Japan; 2 Pharmaceutical Research Laboratories, Research and Development Headquarters, Lion Corporation, Odawara 256-0811, Japan

## Abstract

A lipid layer consisting of meibum lipids exists in the tear film and functions in preventing dry eye disease. Although the meibum lipids include diverse lipid classes, the synthesis pathway and role of each class remain largely unknown. Here, we created single and double knockout (KO and DKO, respectively) mice for the two acyl-CoA wax alcohol acyltransferases (*Awat1* and *Awat2*) and investigated their dry eye phenotypes and meibum lipid composition. *Awat2* KO and DKO mice exhibited severe dry eye with meibomian gland dysfunction, whereas *Awat1* KO mice had mild dry eye. In these mice, specific meibum lipid classes were reduced: (*O*-acyl)-ω-hydroxy fatty acids and type 1ω wax diesters in *Awat1* KO mice, wax monoesters and types 1ω and 2ω wax diesters in *Awat2* KO mice, and most of these in DKO mice. Our findings reveal that Awat1 and Awat2 show characteristic substrate specificity and together produce diverse meibum lipids.

## Highlights

• *Awat2* knockout mice exhibit severe dry eye phenotypes
• *Awat1* knockout mice exhibit mild dry eye phenotypes
• Awat2 is responsible for the production of wax monoesters and type 2ω wax diesters
• Awat1 is involved in (*O*-acyl)-ω-hydroxy fatty acid production

## Graphical abstract

**Figure:**
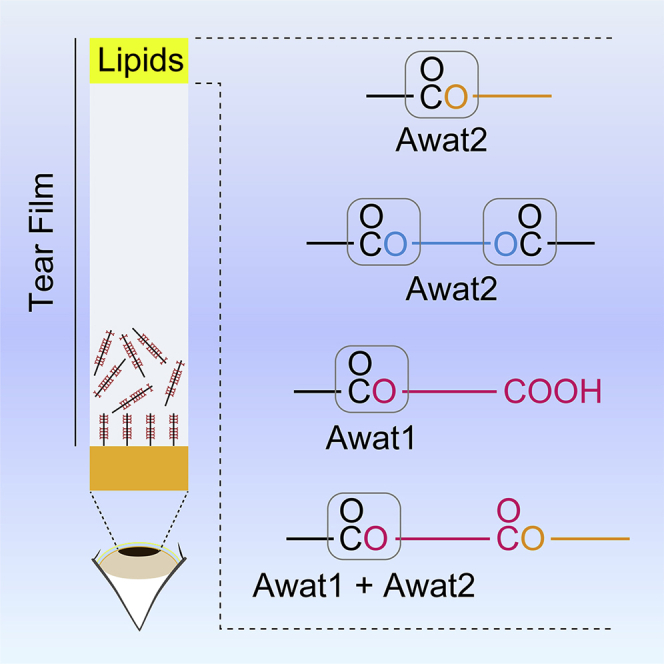

## Introduction

**Figure 1:**
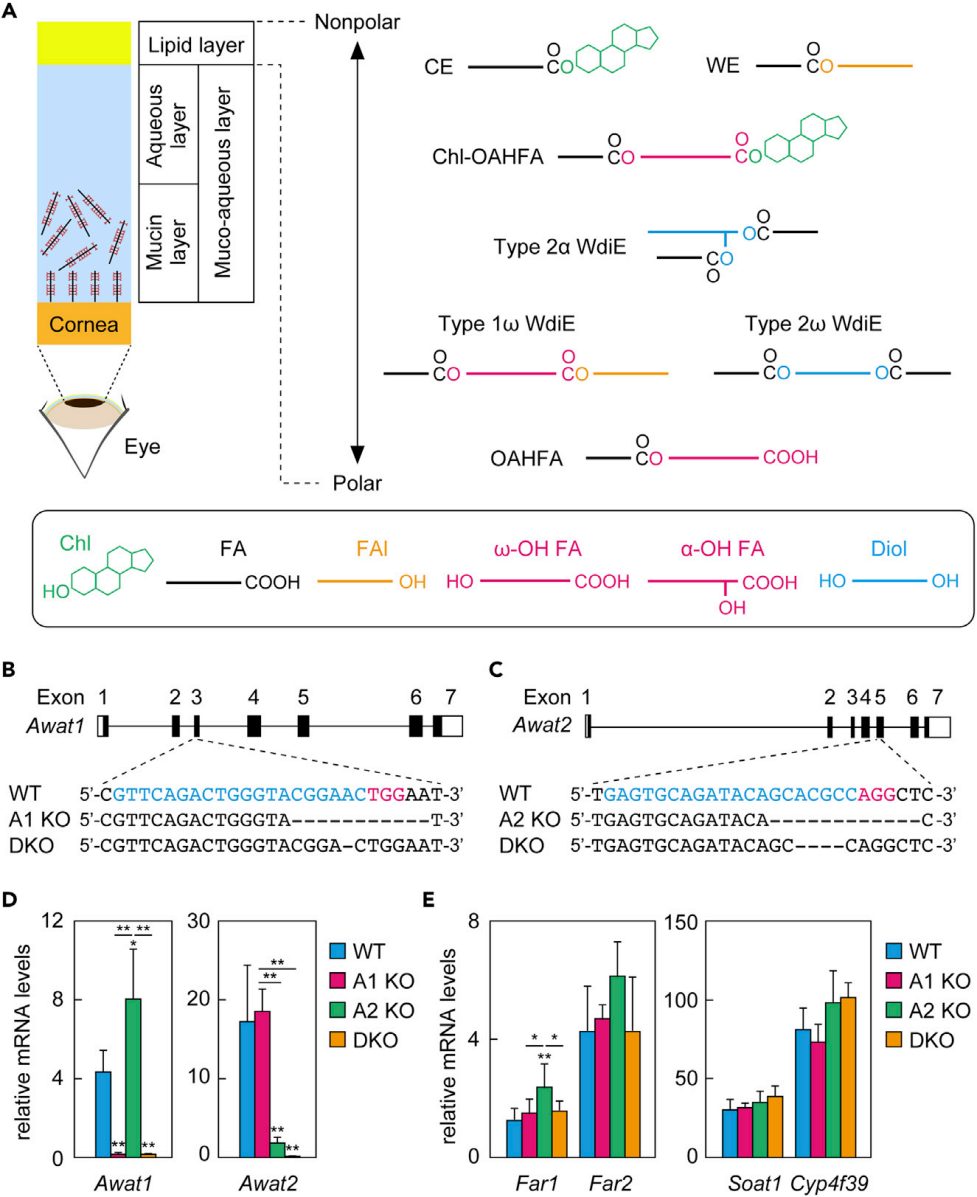
Generation of *Awat1* KO, *Awat2* KO, and *Awat1 Awat2* DKO mice (A) Schematic illustration of the eye, cornea, and tear film and the simplified structures of the major meibum lipids. (B and C) The gene structures (black, coding regions; white, untranslated regions) of *Awat1* (B) and *Awat2* (C) and the nucleotide sequences around the guide RNA target sequences. The blue and red nucleotides in the WT sequence represent the target sequence and the protospacer-adjacent motif sequence, respectively. (D and E) Total RNAs were prepared from the meibomian glands of 6-week-old WT (n = 4), *Awat1* KO (n = 4), *Awat2* KO (n = 4), and DKO (n = 4) mice and subjected to real-time quantitative RT-PCR using specific primers for *Awat1* (D), *Awat2* (D), *Far1* (E), *Far2* (E), *Soat1* (E), *Cyp4f39* (E), or the housekeeping gene *Hprt* (D and E). Values presented are mean (±SD) quantities of each mRNA relative to those of *Hprt*. Significant differences from the WT mice (asterisks above columns) and among the mutants (asterisks above horizontal lines) are indicated (∗p < 0.05; ∗∗p < 0.01; Tukey's test). A1 KO, *Awat1* KO; A2 KO, *Awat2* KO.

The tear film that covers the cornea plays an essential role in the functional maintenance of the cornea. The tear film consists of the tear film lipid layer (TFLL), an aqueous layer, and a mucin layer, in order from outside to inside (Figure 1A) (Bron et al., 2004; Green-Church et al., 2011). Of these, the mucin and aqueous layers together are referred to as the muco-aqueous layer since some mucins may diffuse into the aqueous layer (Craig et al., 2017a; Willcox et al., 2017). The roles of the TFLL include preventing water evaporation from the aqueous layer, reducing the surface tension of the tears, maintaining appropriate tear viscoelasticity, and smoothing the corneal surface (Bron et al., 2004; Cwiklik, 2016; Georgiev et al., 2014; Knop et al., 2011). The mucin layer functions to retain the tear film on the cornea (Bron et al., 2004; Gipson, 2016; Green-Church et al., 2011; Willcox et al., 2017). The aqueous layer is responsible for supplying oxygen and nutrients to the cornea and preventing infection (Garg and Zhang, 2017; Willcox et al., 2017). The components of each layer are secreted by different glands or cells: most of the TFLL lipid components are secreted by the meibomian glands; the aqueous layer by the lacrimal glands; and the mucin layer by goblet cells or corneal epithelial cells (Garg and Zhang, 2017; Gipson, 2016; Knop et al., 2011). The meibomian glands, which are specialized sebaceous glands, are located behind the eyelids, and the lipids they secrete are collectively called meibum lipids (Butovich, 2017; Knop et al., 2011).

According to the 2017 report of the second Dry Eye Workshop organized by the Tear Film and Ocular Surface Society, dry eye disease is a multifactorial disease of the ocular surface characterized by a loss of homeostasis of the tear film and accompanied by ocular symptoms in which tear film instability and hyperosmolarity, ocular surface inflammation and damage, and neurosensory abnormalities play etiological roles (Craig et al., 2017a, 2017b). Dry eye disease is divided into two main categories—aqueous-deficient dry eye and evaporative dry eye (EDE)—and both often occur together (Wolffsohn et al., 2017). The most common cause of EDE is meibomian gland dysfunction (MGD), which accounts for 65–87% of dry eye disease, depending on region and study (Horwath-Winter et al., 2003; Lemp et al., 2012; Shimazaki et al., 1995; Wolffsohn et al., 2017). The pathology of MGD includes obstruction of the meibomian gland orifice, gland atrophy and dropout, and changes in the nature of the meibum lipids (Bron et al., 2017; Tomlinson et al., 2011). Obstruction of the meibomian gland orifice is mainly caused by hyperkeratinization of the duct epithelium (Knop et al., 2011). Meibum secretions are clear under normal conditions, but in patients with MGD, they are cloudy and granular, with the color varying from whitish gray to yellow, or toothpaste like (Tomlinson et al., 2011). It is unclear whether these abnormal properties of the meibum secretions are due to changes in meibum lipid composition.

The meibum lipids include various lipid classes. The major constituents are cholesteryl esters (CEs) and wax (mono)esters (WEs), the sum of which is considered to account for 60–92% of total meibum lipids, depending on the study (Butovich, 2017; Chen et al., 2013; Lam et al., 2011). Other minor lipid classes included in the meibum lipids are (*O*-acyl)-ω-hydroxy fatty acids (OAHFAs, 1–5%), wax diesters (WdiEs, ∼7%), and cholesteryl-OAHFAs (Chl-OAHFAs, ∼3%) (Butovich, 2017; Chen et al., 2013) (Figure 1A). CEs and WEs are the most hydrophobic lipids in mammals. It has been proposed that the amphiphilic OAHFAs, which form an amphiphilic lipid sublayer, intervene between the CEs/WEs (nonpolar lipid sublayer) and the aqueous phase to maintain a stable TFLL above the aqueous layer (Butovich, 2009). However, considering that the tear film also contains a variety of meibum lipid classes, such as WdiEs and Chl-OAHFAs, that exhibit intermediate polarity between CEs/WEs and OAHFAs, the actual TFLL is likely to be more complex than the simple two sublayer model (nonpolar and amphiphilic lipid sublayers). Therefore, we propose a model in which the formation of a lipid polarity gradient by a variety of meibum lipids facilitates stable retention of the TFLL on the aqueous layer (Miyamoto et al., 2020b).

CEs are composed of cholesterol (Chl) and a fatty acid (FA) and WEs of an FA and a fatty alcohol (FAl), both via an ester bond (Figure 1A). Based on the carbon (C) chain length, FAs and FAls are classified into long chain (C11–20), very long chain (VLC; ≥C21), and ultra long chain (ULC; ≥C26) (Kihara, 2012, 2016). The FA moieties of the WEs in meibum lipids are long chain (mainly C18:1 in humans and C16:1 in mice), while the FAl moieties are mostly VLC or ULC (C24–C30) (Butovich, 2017; Sassa et al., 2018; Tanno et al., 2021). OAHFAs consist of an ω-hydroxy (ω-OH) ULCFA (C30–C36) and a long-chain FA (Figure 1A) (Butovich, 2009, 2017; Miyamoto et al., 2020b). WdiEs are classified into type 1 (FA + hydroxy FA + FAl) and type 2 (2 FAs + fatty diol), which are further divided into α-type and ω-type depending on the position of the hydroxyl groups (Figure 1A). The meibum lipids include at least type 1ω, type 2α, and type 2ω WdiEs (Miyamoto et al., 2020b).

To elucidate the role of each meibum lipid in tear film formation and dry eye prevention, analyses of knockout (KO) mice of specific genes involved in meibum lipid synthesis are useful. For example, KO mice of the sterol *O*-acyltransferase 1 *Soat1* gene, which is involved in CE synthesis, exhibit meibomian gland atrophy (Yagyu et al., 2000), revealing the essential role of CEs in meibomian gland maintenance. KO mice of the FA ω-hydroxylase gene *Cyp4f39* (*CYP4F22* in humans), which is responsible for the production of ω-hydroxy lipids (OAHFAs, Chl-OAHFAs, and type 1ω and type 2ω WdiEs), show decreased tear film stability and mild obstruction of the meibomian gland orifice (Miyamoto et al., 2020b). FA elongases catalyze the rate-limiting step of the FA elongation cycle, which elongates acyl-CoA by two carbons per cycle (Kihara, 2012, 2016). Seven FA elongase isoenzymes (ELOVL1–7) with different substrate specificities are found in mammals (Kihara, 2012, 2016; Ohno et al., 2010). Disruption of *Elovl1*, which is responsible for the production of saturated and monounsaturated C22–C26 FAs, causes shortening of CEs and WEs, leading to increased water evaporation from the tear film and age-dependent corneal opacity (Sassa et al., 2018; Watanabe et al., 2021). In addition, mutant mice of *Elovl3* and *Elovl4*, whose gene products are active toward C18–C22 and ≥C26 acyl-CoAs, respectively, also show dry-eye-like phenotypes (Butovich et al., 2019; McMahon et al., 2014), indicating that maintaining the proper chain length in meibum lipids is important for normal TFLL function.

The ester bond formation in WEs is catalyzed by acyl-CoA wax alcohol acyltransferases (AWATs). Mammals contain two AWAT isozymes (AWAT1 and AWAT2) (Cheng and Russell, 2004b; Turkish et al., 2005). *In vitro* experiments have shown that they have the capacity to produce at least long-chain FAl-containing WEs (Cheng and Russell, 2004b; Turkish et al., 2005). Recently, it was reported that *Awat2* KO mice exhibit a dry eye phenotype accompanied by obstruction of the meibomian gland orifice (Widjaja-Adhi et al., 2020). In these mice, the abundance of WEs was greatly reduced relative to wild-type (WT) mice. However, the effect of *Awat2* disruption on the production of other ester-bond-containing meibum lipids, such as WdiEs, OAHFAs, and Chl-OAHFAs, remains largely unclear. Furthermore, the contribution of *Awat1* to the production of WEs and other meibum lipids is completely unknown. In the present study, we generated *Awat1* KO, *Awat2* KO, and *Awat1 Awat2* double KO (DKO) mice and used them to examine the contribution of *Awat1* and *Awat2* to the production of meibum lipids, as well as their roles in dry eye prevention.

## Results

### Generation of *Awat1* KO, *Awat2* KO, and *Awat1 Awat2* DKO mice

To reveal the contribution of *Awat1* and *Awat2* to the production of WEs and other ester-bond-containing meibum lipids, *Awat1* KO, *Awat2* KO, and *Awat1 Awat2* DKO mice were generated using the CRISPR/Cas9 system. Since the *Awat1* and *Awat2* genes are in close proximity to each other on the same chromosome (X), the creation of *Awat1 Awat2* DKO mice by crossing *Awat1* KO mice and *Awat2* KO mice had been predicted to be difficult. Therefore, we tried to disrupt both genes simultaneously by co-injecting *Awat1* and *Awat2* guide RNAs into the same fertilized mouse eggs. The resulting mice included single KO mice with a mutation in one of the genes (*Awat1* KO and *Awat2* KO) and DKO mice with mutations in both genes (*Awat1 Awat2* DKO). The *Awat1* KO mice had an 11 bp deletion in exon 3 of *Awat1*, and the *Awat2* KO mice had a 12 bp deletion in exon 5 of *Awat2* (Figures 1B, 1C, and S1). The DKO mice had a 1 bp deletion in exon 3 of *Awat1* and a 4 bp deletion in exon 5 of *Awat2*. All the *Awat* KO/DKO mice were produced from Mendelian inheritance and grew to adulthood in good health.

Quantitative real-time reverse transcription (RT)-polymerase chain reaction (PCR) was performed to examine the effect of deletion of each of the *Awat* genes on its own expression and that of the other one. *Awat1* mRNA expression was upregulated 1.8-fold in *Awat2* KO mice compared to WT mice (Figure 1D). *Awat2* mRNA expression was similar in WT and *Awat1* KO mice. The expression levels of both the genes with the deletion mutation were reduced, probably due to nonsense-mediated mRNA decay. Next, the expression levels of other meibum-lipid-related genes in the mutant mice were examined. The FAls in WEs are produced from acyl-CoAs by fatty acyl-CoA reductases (FARs). In mammals, there are two FARs (FAR1 and FAR2) (Cheng and Russell, 2004a), and one or both of these may be involved in the production of FAls in meibum lipids. *Far1* expression was slightly increased in *Awat2* KO mice compared to WT mice, whereas *Far2* expression was unchanged in all mutant mice (Figure 1E). Neither the expression levels of *Soat1*, which is involved in CE production (Yagyu et al., 2000), nor of the gene for FA ω-hydroxylase, *Cyp4f39*, differed between the WT and mutant mice.

### *Awat1* or *Awat2* deficiency causes MGD dry eye

**Figure 2:**
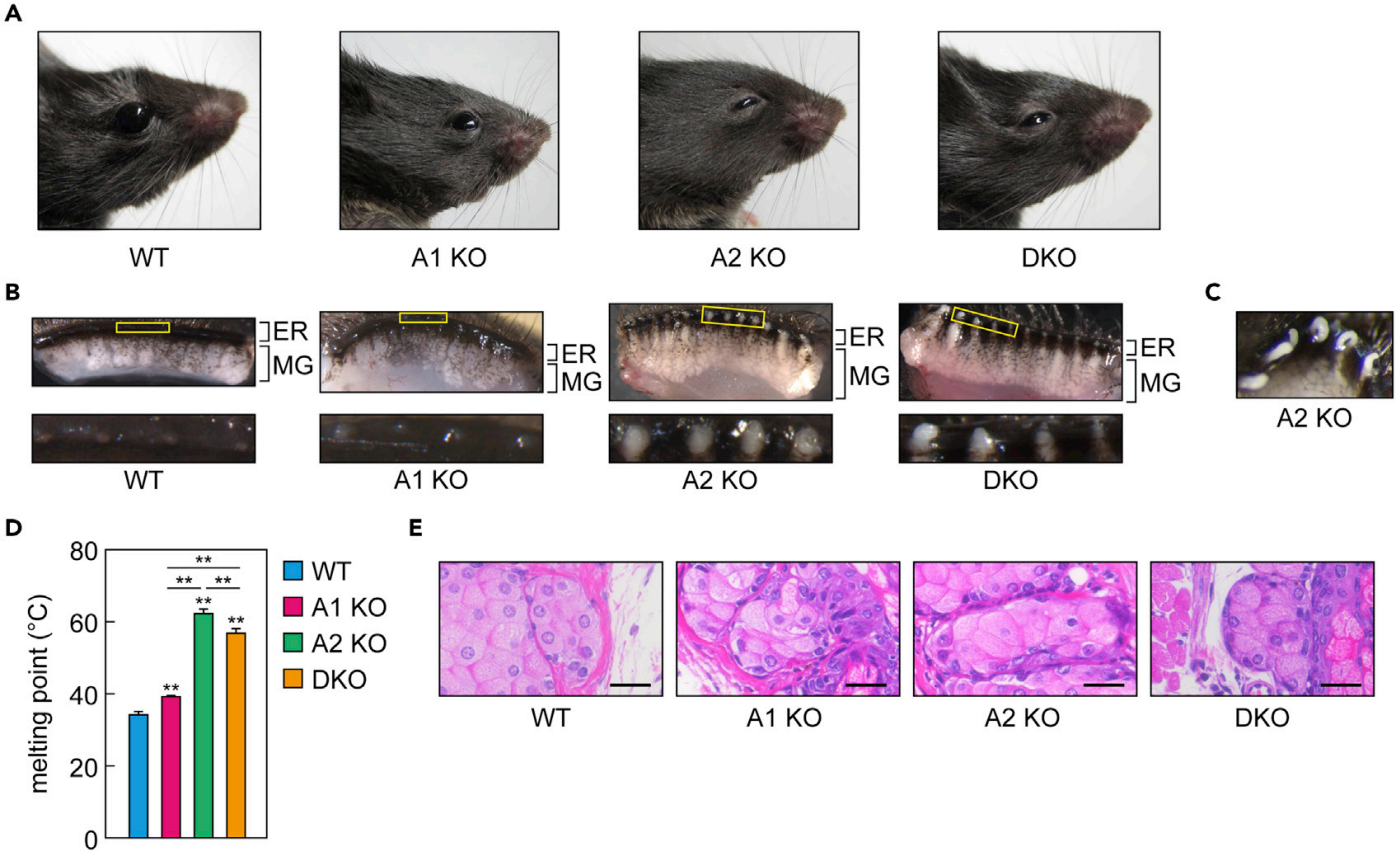
*Awat1* and/or *Awat2* deficiency causes plugging of the meibomian gland orifices (A) Photographs of 6-week-old WT, *Awat1* KO, *Awat2* KO, and *Awat1 Awat2* DKO mice. (B) Upper eyelids from 6-week-old WT, *Awat1* KO, *Awat2* KO, and DKO mice, photographed under a light microscope. The lower images are magnified views of the yellow rectangles in the upper images and show the meibomian gland (MG) orifices. ER, eyelid rim. (C) The upper eyelid of a 6-week-old *Awat2* KO mouse after the meibomian glands were subjected to pressure, causing them to extrude meibum from the orifices. (D) The melting point of meibum lipids extruded from the meibomian gland orifices was measured in 6-week-old WT (n = 3), *Awat1* KO (n = 3), *Awat2* KO (n = 3), and DKO (n = 3) mice. Values presented are means ± SD. Significant differences from WT mice (asterisks above columns) and among the mutants (asterisks above horizontal lines) are indicated (∗∗p < 0.01; Tukey's test). (E) Paraffin sections of the meibomian glands in 6-week-old WT, *Awat1* KO, *Awat2* KO, and DKO mice were stained with hematoxylin and eosin. The bright-field images were photographed under a light microscope. Scale bar, 25 μm. A1 KO, *Awat1* KO; A2 KO, *Awat2* KO.

The eyes of the WT mice were typically held wide open, whereas those of the *Awat1* KO mice were held slightly closed and those of the *Awat2* KO and DKO mice were held more closed (Figure 2A). Plugging is often observed at the orifice of the meibomian glands in patients with MGD dry eye (Bron et al., 2017; Tomlinson et al., 2011). No such plugging was observed in the WT mice, but the orifice was blocked by a whitish plug in all the 6-week-old mutant mice (Figure 2B). The plugging substance in the *Awat2* KO and DKO mice was solid, while that in the *Awat1* KO mice was semiliquid. A paste-like meibum was extruded when the meibomian glands of the *Awat2* KO mice were subjected to pressure (Figure 2C). The melting points of the meibum lipids in *Awat2* KO (62°C) and DKO mice (57°C) were much higher than those in WT mice (34°C), whereas in *Awat1* KO mice, it was only slightly higher (39°C) (Figure 2D). Hematoxylin and eosin staining revealed no morphological abnormalities in the acini of the meibomian glands in any of the mutant mice (Figure 2E).

The meibomian glands were enlarged in the *Awat2* KO and DKO mice but not in the WT or *Awat1* KO mice, probably due to the inhibition of meibum secretion by the firm plugging substance (Figure 2B). Plugging of the meibomian gland orifices in *Awat* KO/DKO mice was already observed at 3 weeks of age, and plugging similar to that at 6 weeks of age was observed in old mice (22–26 months of age; Figure S2). Weak age-dependent meibomian gland degeneration was observed in all old mice, including WT animals. However, *Awat* gene-disruption-dependent meibomian gland degeneration did not seem to occur, even at 22–26 months of age, at least macroscopically.

**Figure 3:**
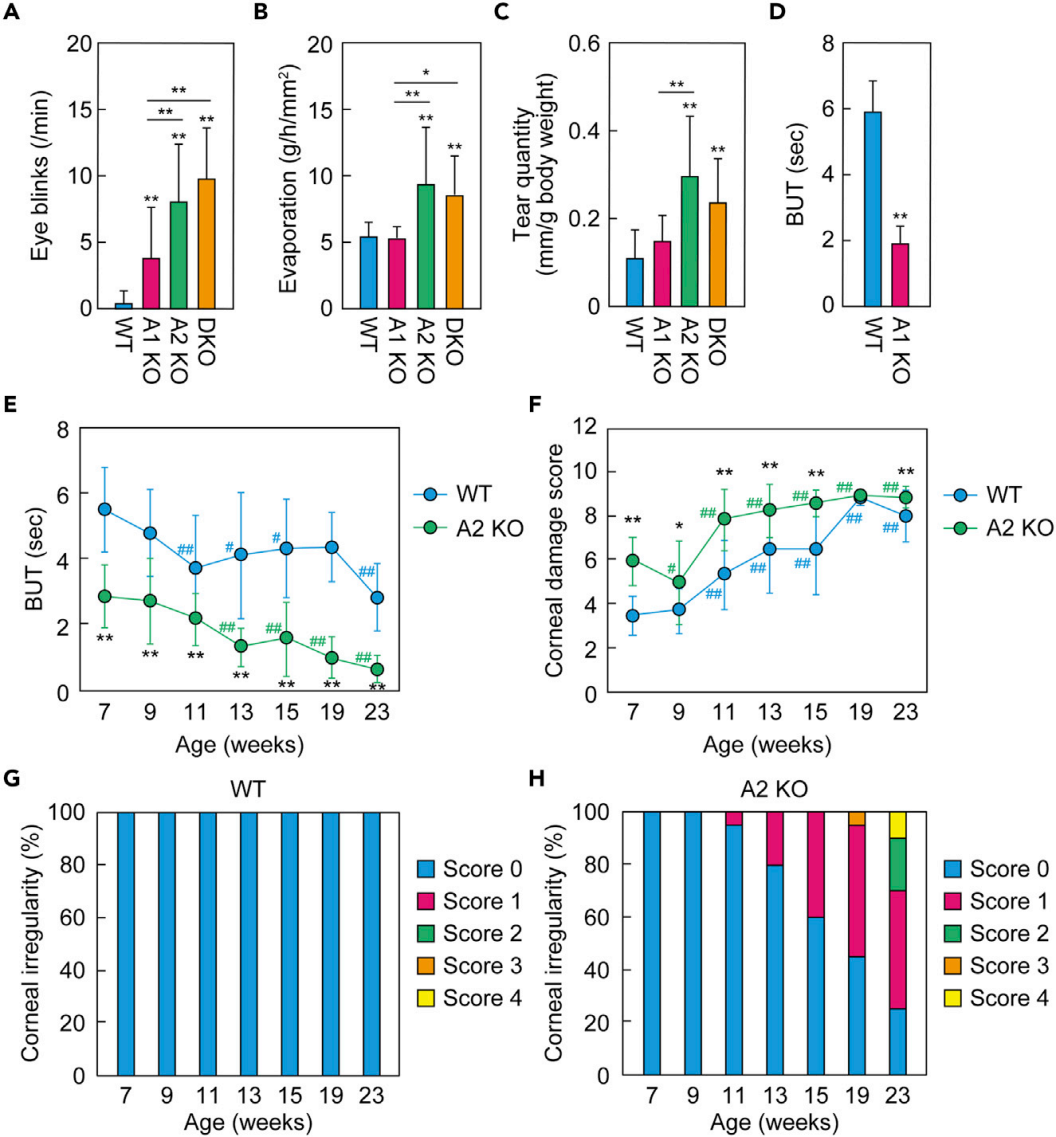
*Awat1* or *Awat2* deficiency causes dry eye phenotypes (A–C) Blink frequency (A), water evaporation from the ocular surface (B), and tear quantity (C) were measured in 6-week-old WT, *Awat1* KO, *Awat2* KO, and *Awat1 Awat2* DKO mice. Values presented are means ± SD. The number of mice of each line examined was as follows: WT, n = 30 (A) or n = 28 (B and C); *Awat1* KO, n = 14 (A) or n = 11 (B and C); *Awat2* KO, n = 14 (A and C) or n = 13 (B); DKO, n = 17 (A) or n = 16 (B and C). Significant differences from the WT mice (asterisks above columns) and among the mutants (asterisks above horizontal lines) are indicated (∗∗p < 0.01; Tukey-Kramer test). (D) BUT was measured in 6-week-old WT (n = 3) and *Awat1* KO (n = 3) mice. Values presented are means ± SD. Significant differences from the WT mice are indicated (∗∗p < 0.01; Student's *t*-test). (E–H) BUT (E), corneal damage score (F), and corneal surface irregularity score (G and H) were measured in WT (n = 10) (E–G) and *Awat2* KO (n = 10) (E, F, and H) mice. Experiments were performed from the age of 7 weeks to 23 weeks, and measurements were performed on both eyes. (E and F) Values presented are means ± SD, and significant differences from WT mice of the same age (∗p < 0.05; ∗∗p < 0.01; Student's *t*-test) and from mice of the same genotype at 7 weeks old (#p < 0.05; ##p < 0.01; Dunnett's test) are indicated. (G and H) The proportion of the mice that had each score (0–4) at each age. A1 KO, *Awat1* KO; A2 KO, *Awat2* KO.

Increases in blinking frequency in EDE model mice have been reported (Miyamoto et al., 2020b; Sassa et al., 2018). The blinking frequency in the WT mice was 0.4 per min but that in the *Awat2* KO and DKO mice was much higher (8.1 and 9.8 per min, respectively; Figure 3A). The blinking frequency in the *Awat1* KO mice was 3.8 per min, which was significantly higher than that in the WT mice. Although neither water evaporation from the eyes nor the tear fluid volume in *Awat1* KO mice differed significantly from those in WT mice, they were higher in *Awat2* KO and DKO mice than in WT mice (water evaporation: 1.7-fold in *Awat2* KO mice, 1.6-fold in DKO mice; tear fluid volume: 2.7-fold in *Awat2* KO mice, 2.2-fold in DKO mice; Figures 3B and 3C). The greater quantities of tear fluid may be a compensation for the greater water evaporation from the eyes. We next measured tear breakup time (BUT), an indicator of the stability of the tear film, with lower values indicating poorer stability. *Awat1* KO mice exhibited lower BUT values than WT mice (Figure 3D). *Awat2* KO mice also showed low BUT values, which decreased with age (Figure 3E). At all timepoints measured (7–23 weeks of age), the BUT in *Awat2* KO mice was shorter than that in WT. We then examined other dry eye phenotypes, such as corneal damage and corneal irregularity, in *Awat2* KO mice. Corneal damage scores were higher at 11–23 weeks than those at 7 weeks in both the WT and *Awat2* KO mice and had reached almost the maximum possible score by 11 weeks in the *Awat2* KO mice (Figure 3F). The *Awat2* KO mice scored higher than the WT mice at all timepoints except 19 weeks. With respect to corneal irregularity, no abnormality was observed in the WT mice at any time point measured (Figure 3G). In contrast, irregularity was observed in the *Awat2* KO mice from the age of 11 weeks and increased until 23 weeks (Figure 3H). Thus, the *Awat2* KO mice exhibited a time-dependent progression of dry eye.

### *Awat2* is involved in WE production

**Figure 4:**
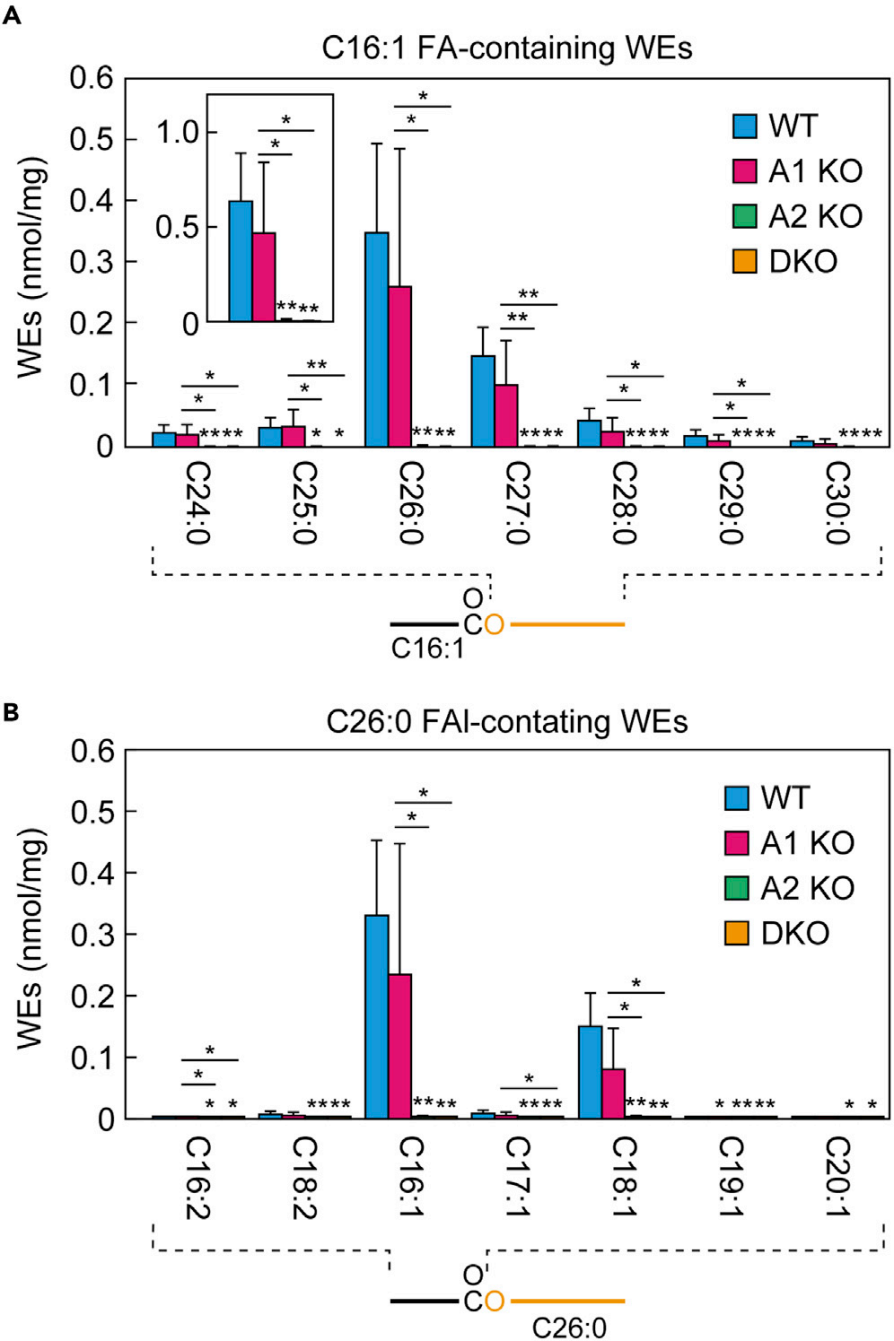
*Awat2* is involved in WE production Lipids were extracted from the meibomian glands of 6-week-old WT (n = 5), *Awat1* KO (n = 5), *Awat2* KO (n = 5), and *Awat1 Awat2* DKO (n = 5) mice, and WEs were analyzed using LC-MS/MS. (A) Quantities of the major WEs (nmol/mg tissue) composed of a C16:1 FA moiety and a saturated FAl moiety with a chain length of C24–C30. Inset shows the total quantity of WEs composed of a C16:1 FA moiety and a saturated FAl moiety with a chain length of C16–C36. (B) Quantities of WEs (nmol/mg tissue) containing one of the indicated FA moieties and a C26:0 FAl moiety. Values presented are means ± SD, and significant differences from the WT mice (asterisks above columns) and among the mutants (asterisks above horizontal lines) are indicated (∗p < 0.05; ∗∗p < 0.01; Tukey's test). The simplified structure of a WE with the analyzed moiety (FAl or FA) indicated is shown below each graph. Data for all WEs measured are provided in Table S1. A1 KO, *Awat1* KO; A2 KO, *Awat2* KO.

To investigate the contribution of *Awat1* and *Awat2* to WE production, we measured the quantities of WEs in the meibum lipids using liquid chromatography (LC)-tandem mass spectrometry (MS/MS). WEs consist of an FA and an FAl, and the FA moiety in the majority of mouse meibum lipids is C16:1 FA (Butovich, 2017). The FAl moiety of the WEs with C16:1 FA in the WT mouse meibum lipids was predominantly C24:0–C30:0, with the most common being C26:0 (Figure 4A and Table S1). Consistent with the previous report (Butovich, 2017), the most abundant FA moiety of the WEs containing C26:0 FAl was C16:1, followed by C18:1 (Figure 4B). WEs containing other monounsaturated FA or saturated FA were present in trace amounts or almost absent (Figure 4B and Table S1). The quantities of WEs in the *Awat1* KO mice were similar to those in the WT mice for all WE species, irrespective of FA/FAl combination (Figures 4A and 4B and Table S1). In contrast, in the *Awat2* KO and DKO mice, almost all WE species were absent. These results indicate that *Awat2* alone is responsible for WE production in the meibomian glands.

### *Awat1* is involved in OAHFA production

**Figure 5:**
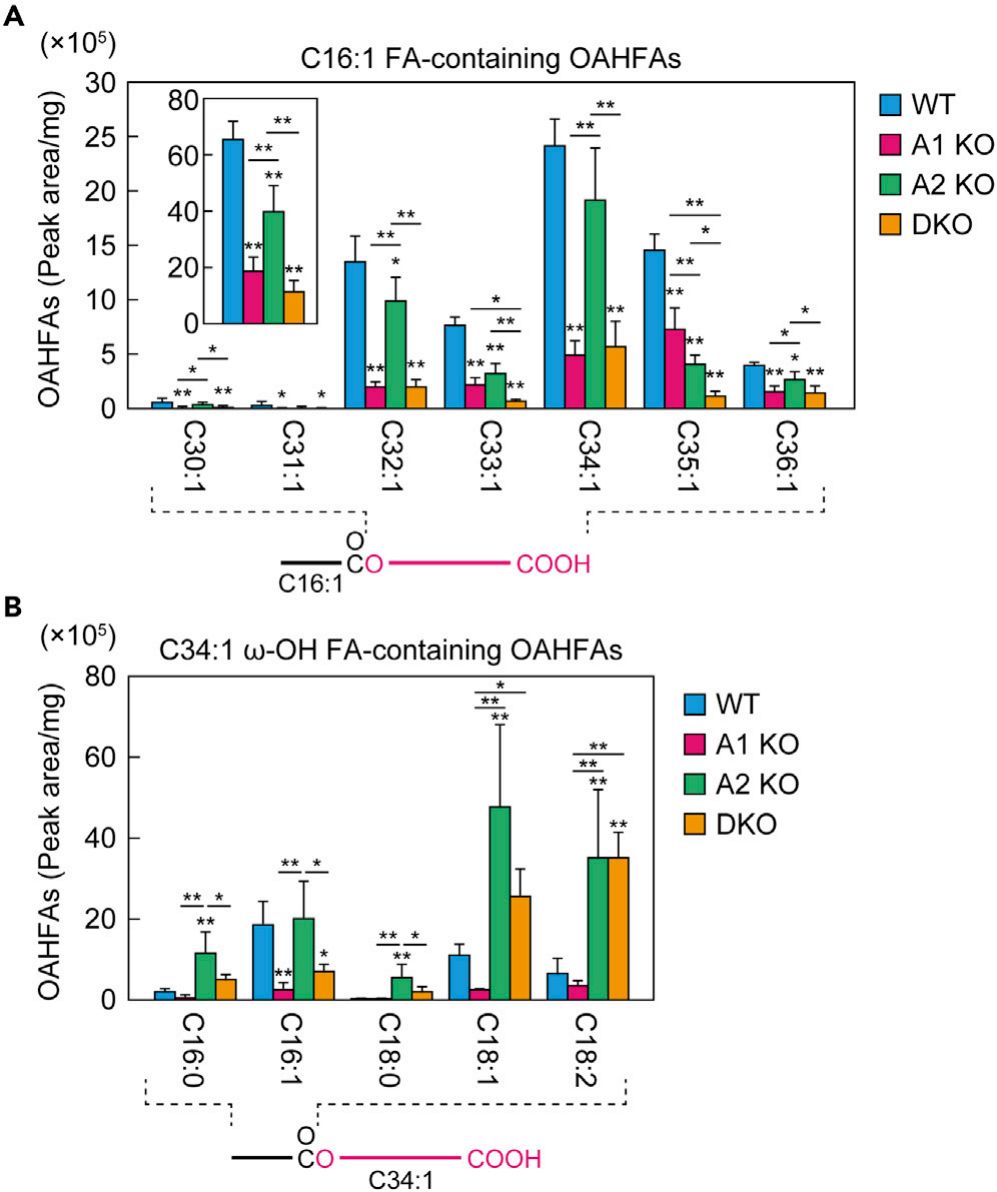
*Awat1* is involved in OAHFA production Lipids were extracted from the meibomian glands of 6-week-old WT (n = 4), *Awat1* KO (n = 4), *Awat2* KO (n = 4), and *Awat1 Awat2* DKO (n = 4) mice. After derivatization with *N*-(4-aminomethylphenyl)pyridinium, OAHFAs were analyzed using LC-MS/MS. (A) Quantities of OAHFAs (peak area/mg tissue) composed of a C16:1 FA moiety and a monounsaturated ω-OH FA moiety with a chain length of C30–C36. Inset shows the total quantities of OAHFAs composed of a C16:1 FA moiety and a monounsaturated ω-OH FA moiety with a chain length of C16–C36. (B) Quantities of OAHFAs (peak area/mg tissue) containing one of the indicated FA moieties and a C34:1 ω-OH FA moiety. Values presented are means ± SD, and significant differences from the WT mice (asterisks above columns) and among the mutants (asterisks above horizontal lines) are indicated (∗p < 0.05; ∗∗p < 0.01; Tukey's test). The simplified structure of an OAHFA with the analyzed moiety (ω-OH FA or FA) indicated is shown below each graph. Data for all OAHFAs measured are provided in Table S2. A1 KO, *Awat1* KO; A2 KO, *Awat2* KO.

OAHFAs are composed of a long-chain FA (mainly C16:1, C18:1, and C18:2) and an ω-OH ULCFA (mainly monounsaturated C30–C36) (Butovich, 2009, 2017; Miyamoto et al., 2020b). However, the acyltransferase that catalyzes the formation of the ester bond between them has not been identified. In the present study, to investigate the contribution of *Awat1* and *Awat2* to OAHFA production, we measured the quantities of OAHFAs using LC-MS/MS. We first measured the OAHFAs with a C16:1 FA in the WT mouse meibomian glands and found that the most abundant one had C34:1 as the ω-OH FA moiety, and the next most abundant had C35:1 (Figure 5A and Table S2). The majority of the ω-OH FA moieties were monounsaturated, and only trace quantities of the OAHFAs had saturated ω-OH FAs (Table S2). The abundance of OAHFAs in the *Awat1* KO mice was reduced for all major species (C32:1–C36:1) compared to WT mice, and the total quantity was 29% of that in the WT mice (Figure 5A and Table S2). OAHFA levels in *Awat2* KO mice were slightly reduced for some molecular species relative to WT mice and those in DKO mice were similar to those in *Awat1* KO mice for most species. We then measured the quantities of OAHFAs with different FA moieties (C16:0, C16:1, C18:0, C18:1, or C18:2) and C34:1 as the ω-OH FA moiety. The quantities of all of these OAHFAs in *Awat1* KO mice were reduced relative to WT mice, although this difference was only significant in the case of those containing a C16:1 FA (Figure 5B and Table S2). However, the quantities of all OAHFAs except for those containing a C16:1 FA were increased in *Awat2* KO mice. Although the levels of most of the OAHFA species were reduced in DKO mice compared to *Awat2* KO mice, those containing a C18:1 or C18:2 FA remained at high levels in DKO mice, suggesting the involvement of an acyltransferase other than Awat1 or Awat2. These results indicate that *Awat1* plays a major role in OAHFA production.

### *Awat2* is involved in type 2ω WdiE production

**Figure 6:**
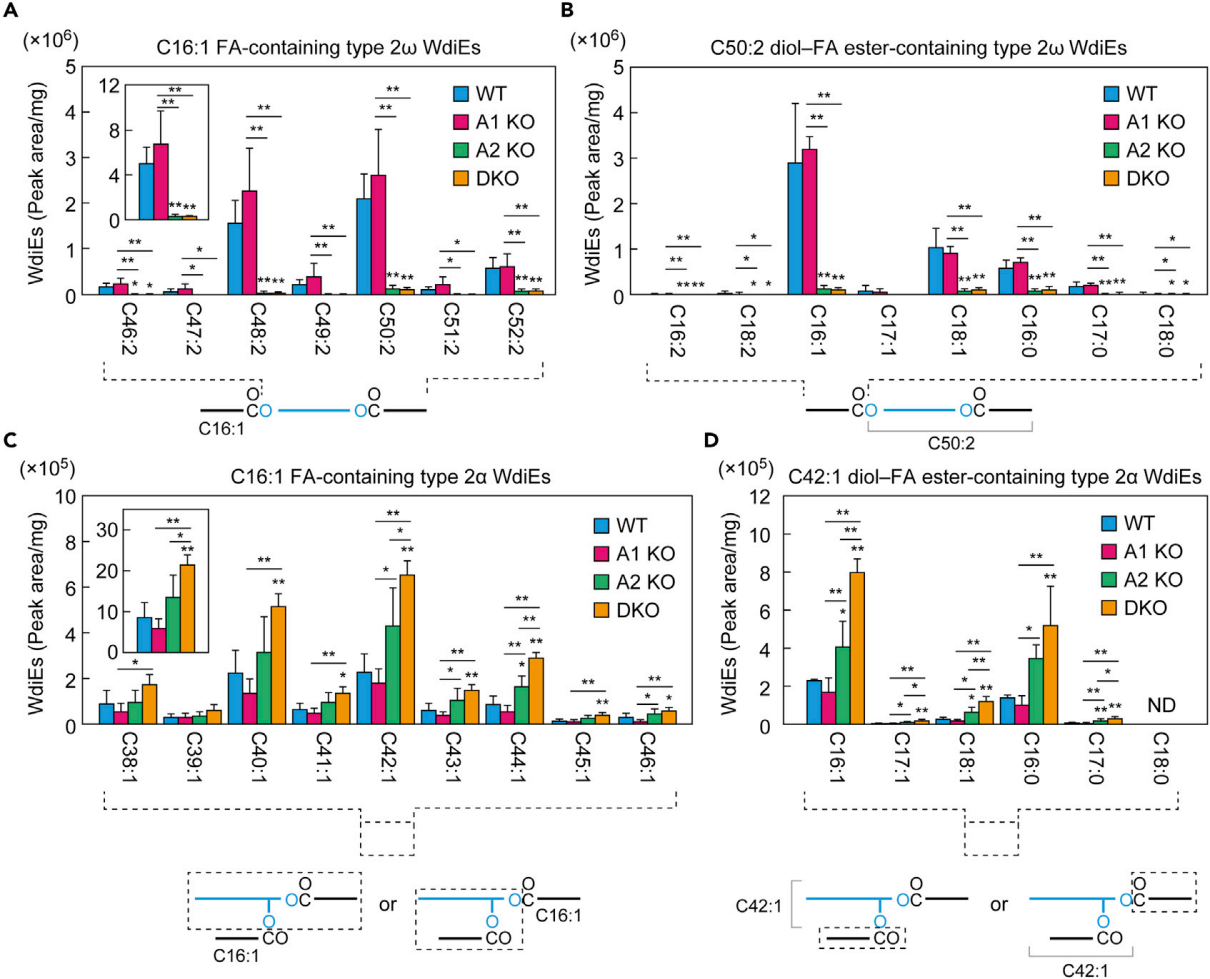
*Awat2* is involved in type 2ω WdiE production Lipids were extracted from the meibomian glands of 6-week-old WT (n = 4), *Awat1* KO (n = 4), *Awat2* KO (n = 4), and *Awat1 Awat2* DKO (n = 4) mice, and type 2ω WdiEs (A and B) and type 2α WdiEs (C and D) were analyzed using LC-MS/MS. (A) Quantities of type 2ω WdiEs (peak area/mg tissue) composed of a C16:1 FA moiety and a di-unsaturated diol-FA ester moiety with a chain length of C46–C52. Inset shows the total quantities of type 2ω WdiEs composed of a C16:1 FA moiety and a di-unsaturated diol-FA ester moiety with a chain length of C32–C54. (B) Quantities of type 2ω WdiEs (peak area/mg tissue) composed of one of the indicated FA moieties and a C50:2 diol-FA ester moiety. (C) Quantities of type 2α WdiEs (peak area/mg tissue) composed of a C16:1 FA moiety and a monounsaturated diol-FA ester moiety with a chain length of C38–C46. Inset shows the total quantities of type 2α WdiEs composed of a C16:1 FA moiety and a monounsaturated diol-FA ester moiety with a chain length of C32–C54. (D) Quantities of type 2α WdiEs (peak area/mg tissue) composed of one of the indicated FA moieties and a C42:1 diol-FA ester moiety. Values presented are means ± SD, and significant differences from the WT mice (asterisks above columns) and among the mutants (asterisks above horizontal lines) are indicated (∗p < 0.05; ∗∗p < 0.01; Tukey's test). The simplified structure of a WdiE with the analyzed moiety (diol-FA ester or FA) indicated is shown below each graph. Data for all type 2ω and 2α WdiEs measured are provided in Tables S3 and S4, respectively. A1 KO, *Awat1* KO; A2 KO, *Awat2* KO; ND, not detected.

Type 2α or 2ω WdiEs are diesters consisting of two FAs and a diol with a hydroxyl group at the α or ω position, respectively. To investigate whether *Awat1* or *Awat2* is involved in the production of type 2ω WdiEs, we measured the levels of type 2ω WdiE in meibum lipids from the WT and mutant mice using LC-MS/MS. Among the type 2ω WdiEs containing a C16:1 FA from the WT mice, C50:2 was the most abundant diol-FA ester moiety (in this moiety, the sum of the chain length and the degree of unsaturation of the diol plus the other long-chain FA is C50:2), followed by C48:2 (Figure 6A and Table S3). Although type 2ω WdiEs containing di-unsaturated diol-FA esters were predominant in the WT meibum lipids, species containing monounsaturated, tri-unsaturated, and saturated diol-FA esters were also present, in this order of abundance (Table S3). With respect to the FA composition of type 2ω WdiEs containing a C50:2 diol-FA ester, C16:1 was the most abundant, followed by C18:1 (36% of the quantity containing a C16:1 FA) and C16:0 (20% of the quantity containing a C16:1 FA) (Figure 6B and Table S3). Combining these results with our previous findings (Miyamoto et al., 2020b), the most abundant type 2ω WdiE species in WT mice is a C16:1 FA-C34:1 diol-C16:1 FA conjugate.

The quantities and compositions of type 2ω WdiEs in the *Awat1* KO mice were similar to those in the WT mice (Figures 6A and 6B and Table S3). However, the quantities of these molecules were greatly reduced in both the *Awat2* KO and the DKO mice compared to the WT mice for almost all the molecular species. The total quantity in both of these mutants was ∼6.4% of that in the WT mice. These results indicate that *Awat2* is responsible for the synthesis of type 2ω WdiEs.

Next, we examined the type 2α WdiE levels in the meibum lipids of the WT and mutant mice using LC-MS/MS. In the WT mice, type 2α WdiEs containing a C38:1-C44:1 diol-FA ester and a C16:1 FA were the predominant species (Figures 6C and 6D and Table S4). The quantities of type 2α WdiEs in the *Awat1* KO mice were similar to those in the WT mice, but they were higher in the *Awat2* KO mice and more so in the DKO mice. These increases were probably due to changes in the lipid metabolism flow caused by the decreases in type 2ω WdiEs and/or other meibum lipids. Our results indicate that neither *Awat1* nor *Awat2* is involved in the synthesis of type 2α WdiEs.

### Differential contribution of *Awat1* and *Awat2* to the production of di-unsaturated and tri-unsaturated type 1ω WdiEs

**Figure 7:**
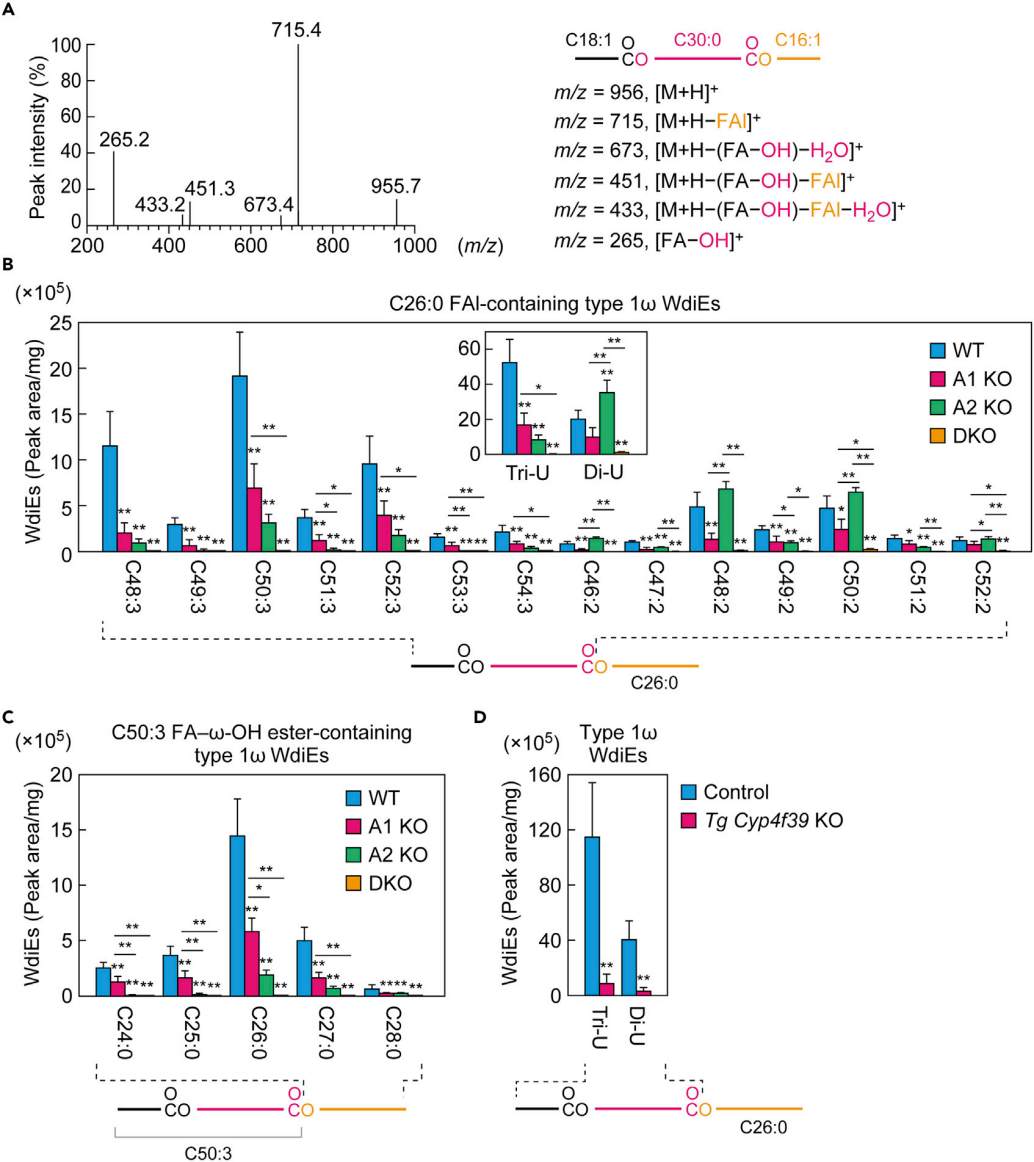
*Awat1* and *Awat2* are differentially involved in di-unsaturated and tri-unsaturated type 1ω WdiE production (A) Product ion scanning of the chemically synthesized type 1ω WdiE standard (C18:1 FA + ω-OH C30:0 FA + C16:1 FAl) was performed using LC-MS/MS, by selecting the [M + H]^+^ ion with *m*/*z* = 955.9 as a precursor. The MS spectrogram and the predicted product ions are shown. The synthesis scheme for the type 1ω WdiE standard is provided in Figure S3. (B–D) Lipids were extracted from the meibomian glands of 6-week-old WT (n = 4), *Awat1* KO (n = 4), *Awat2* KO (n = 4), and *Awat1 Awat2* DKO (n = 4) mice (B and C), or from the meibomian glands of 12-month-old *Tg Cyp4f39* KO (n = 3) and their control (n = 3) mice (D), and type 1ω WdiEs were analyzed using LC-MS/MS. (B) Quantities of type 1ω WdiEs (peak area/mg tissue) composed of a C26:0 ULCFAl moiety and one of the indicated di-unsaturated or tri-unsaturated FA-ω-OH FA ester moieties. Inset shows the total quantities of type 1ω WdiEs composed of a C26:0 ULCFAl moiety and a di-unsaturated or tri-unsaturated FA-ω-OH FA ester moiety with a chain length of C32–C54. (C) Quantities of type 1ω WdiEs (peak area/mg tissue) composed of a saturated FAl moiety with a chain length of C24–C28 and a C50:3 FA-ω-OH FA ester moiety. (D) The total amounts of type 1ω WdiEs (peak area/mg tissue) composed of a C26:0 ULCFAl moiety and a di-unsaturated or tri-unsaturated FA-ω-OH FA ester moiety of C32–C54 chain lengths. Values presented are means ± SD, and significant differences from the WT (B and C) or control mice (D) (asterisks above columns) and among the mutants (asterisks above horizontal lines) are indicated (∗p < 0.05; ∗∗p < 0.01; Tukey's test [B and C] or Student's *t*-test [D]). The simplified structure of a type 1ω WdiE with the analyzed moiety (FA-ω-OH FA ester or FAl) indicated is shown below each graph. Data for all type 1ω WdiEs measured are provided in Tables S5 and S6. A1 KO, *Awat1* KO; A2 KO, *Awat2* KO. Di-U, di-unsaturated; Tri-U, tri-unsaturated.

We previously predicted that peaks showing retention times ∼1 min shorter than those of type 2ω WdiEs in an LC chromatogram represented type 1ω WdiEs, based on the fragmentation pattern obtained by product ion scanning and on their absence in *Tg*(*IVL*-*Cyp4f39*) *Cyp4f39* KO mice (*Tg Cyp4f39* KO), in which the FA ω-hydroxylase gene *Cyp4f39* is disrupted in all tissues except for the epidermis (Miyamoto et al., 2020b). Type 1ω WdiEs are diesters consisting of an FA, an ω-OH FA, and an FAl. In the present study, to verify the validity of our previous peak identification, we chemically synthesized a type 1ω WdiE standard (C18:1 FA + ω-OH C30:0 FA + C16:1 FAl) (Figure S3). We scanned for the product ions of this standard and identified a characteristic product ion as [M + H − FAl]^+^ (*m/z* = 715.4) (Figure 7A). Based on this result, we concluded that the LC-MS/MS method can be used to analyze type 1ω WdiEs by setting [M + H − FAl]^+^ as the product ions to detect in multiple reaction monitoring (MRM) mode. Examination of the type 1ω WdiEs in the meibum lipids of WT mice revealed that the peaks for type 1ω and type 2ω WdiEs overlapped. It is likely that the LC peaks we previously observed (Miyamoto et al., 2020b), which had shorter retention times than those of type 2ω WdiEs, represent isomers of type 1ω WdiEs, such as branched-chain type 1ω WdiEs or other types of WdiEs with a similar structure (e.g., WdiEs with a dicarboxylic acid).

Our new measurement revealed that the most abundant type 1ω WdiE species in WT mouse meibum lipids are composed of a C26:0 FAl and a di-unsaturated or tri-unsaturated FA-ω-OH FA ester with a chain length of C46–C54 (Figures 7B and 7C and Table S5). The total quantity of type 1ω WdiEs containing a tri-unsaturated FA-ω-OH FA ester was 2.6-fold that of those containing a di-unsaturated one (Figure 7B). In the *Awat2* KO mice, the quantities of tri-unsaturated species were greatly reduced (total quantity: 16.2% of WT levels). They were also reduced in the *Awat1* KO mice but more modestly (32.2% of WT levels). Almost no tri-unsaturated species were present in the DKO mice. Thus, both *Awat1* and *Awat2* are involved in the production of the tri-unsaturated species, but the contribution of *Awat2* is greater than that of *Awat1*. However, the contribution of *Awat1* to the production of di-unsaturated species appears to be greater than that of *Awat2*: The total quantity of di-unsaturated species in the *Awat1* KO mice was 49.5% of that in the WT mice but that in the *Awat2* KO mice was slightly higher than that in the WT mice. In the DKO mice, di-unsaturated type 1ω WdiEs were, like the tri-unsaturated species, almost absent (5.4% of WT mice). These results indicate that *Awat1* and *Awat2* contribute differentially to the production of di-unsaturated and tri-unsaturated type 1ω WdiE species.

The lipids we previously incorrectly assigned as type 1ω WdiEs were absent in *Tg Cyp4f39* KO mice (Miyamoto et al., 2020b). In the present study, we measured the quantities of true type 1ω WdiEs in *Tg Cyp4f39* KO mice and found that the production of both di-unsaturated and tri-unsaturated type 1ω WdiEs was greatly impaired (Figure 7D and Table S6).

### *Awat1* and *Awat2* are involved in Chl-OAHFA production

**Figure 8:**
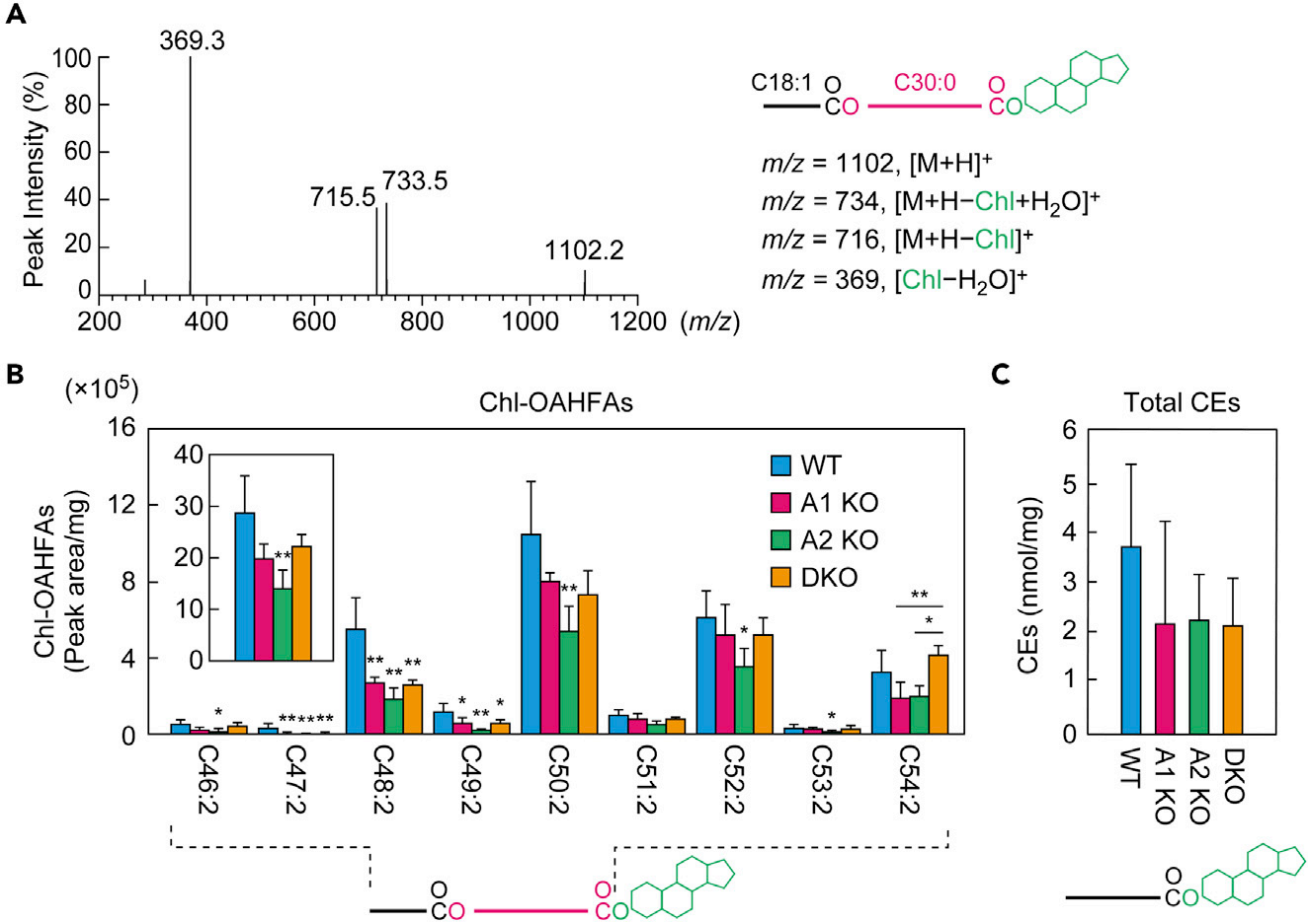
*Awat1* and *Awat2* are partially involved in Chl-OAHFA production (A) Product ion scanning of the chemically synthesized Chl-OAHFA standard (C18:1 FA + ω-OH C30:0 FA + Chl) was performed using LC-MS/MS by selecting the [M + H]^+^ ion with *m*/*z* = 1102.1 as a precursor. The MS spectrogram and the predicted product ions are shown. The synthesis scheme for the Chl-OAHFA standard is provided in Figure S4. (B) Lipids were extracted from the meibomian glands of 6-week-old WT (n = 4), *Awat1* KO (n = 4), *Awat2* KO (n = 4), and *Awat1 Awat2* DKO (n = 4) mice, and Chl-OAHFAs were analyzed using LC-MS/MS. Quantities of Chl-OAHFAs (peak area/mg tissue) containing a di-unsaturated OAHFA moiety with a chain length of C46–C54 are shown. Inset shows the total quantities of Chl-OAHFAs containing a di-unsaturated OAHFA moiety with a chain length of C32–C54. The simplified structure of a Chl-OAHFA with the analyzed moiety (OAHFA) indicated is shown below the graph. (C) Lipids were extracted from the meibomian glands of 6-week-old WT (n = 5), *Awat1* KO (n = 5), *Awat2* KO (n = 5), and DKO (n = 5) mice, and CEs were analyzed using LC-MS/MS. Total quantities of CEs (nmol/mg tissue) containing a saturated or monounsaturated FA moiety with a chain length of C16–C36 are shown. Values presented are means ± SD, and significant differences from the WT mice (asterisks above columns) and among the mutants (asterisks above horizontal lines) are indicated (∗p < 0.05; ∗∗p < 0.01; Tukey's test). Data for all Chl-OAHFAs and CEs measured are provided in Tables S7 and S8, respectively. A1 KO, *Awat1* KO; A2 KO, *Awat2* KO.

In Chl-OAHFAs, a Chl is ester bonded with the carboxyl group of the OAHFA moiety. Since the acyltransferase involved in Chl-OAHFA production is unknown, we investigated the involvement of *Awat1* and *Awat2*. To specifically detect Chl-OAHFAs, we chemically synthesized a Chl-OAHFA standard (C18:1 FA + ω-OH C30:0 FA + Chl) (Figure S4) and subjected it to product ion scanning. We identified a characteristic product ion as [Chl − H_2_O]^+^ (*m/z* = 369.3) (Figure 8A) and thus set [Chl − H_2_O]^+^ as the product ion for the detection of Chl-OAHFAs in MRM mode. In the WT mouse meibum lipids, we detected Chl-OAHFAs containing di-unsaturated OAHFA moieties that predominantly had chain lengths of C46–C54, with the most abundant being C50:2 (Figure 8B and Table S7). Considering our findings regarding the OAHFA composition of the meibum lipids (Figure 5), it is highly likely that the C50:2 OAHFA consists mainly of C16:1 FA and C34:1 ω-OH FA. In both the *Awat1* and *Awat2* KO mice, the abundance of many Chl-OAHFA species was reduced to a greater or lesser extent. The total quantity of Chl-OAHFAs in the *Awat1* KO mice was 69% of that in the WT mice, although this difference was not statistically significant (Figure 8B). The total quantity of Chl-OAHFAs in the *Awat2* KO mice was 49% of that in the WT mice. Although some Chl-OAHFA species were reduced in the DKO mice relative to the WT mice, we observed no reduction relative to the *Awat1* or *Awat2* KO mice; in fact, one species (C54:2) was more abundant than in the single KO mice. The total quantity of Chl-OAHFAs in the DKO mice was 77% of that in the WT mice, but this difference was not statistically significant. These results suggest that both *Awat1* and *Awat2* are involved in Chl-OAHFA production but that an unidentified acyltransferase plays a greater role. We speculate that the expression of this unknown acyltransferase was enhanced in a compensatory manner in the DKO mice. We also measured CE levels in WT and *Awat* KO/DKO mice but observed no significant differences among them (Figure 8C and Table S8).

## Discussion

In this study, we found that each of the mutant mouse lines exhibited characteristic reductions in the abundance of ester-bond-containing meibum lipids. The *Awat1* KO mice exhibited a substantial reduction in OAHFAs and tri-unsaturated type 1ω WdiEs and an approximately 50% reduction in di-unsaturated type 1ω WdiEs (Figures 5 and 7). The *Awat2* KO mice had substantial reductions in WEs, tri-unsaturated type 1ω WdiEs, and type 2ω WdiEs (Figures 4, 6, and 7). Finally, the *Awat1 Awat2* DKO mice exhibited substantial reductions in WEs, C16:1 FA-containing OAHFAs, di-unsaturated and tri-unsaturated type 1ω WdiEs, and type 2ω WdiEs (Figures 4, 5, 6, and 7). The *Awat2* KO and DKO mice showed severe MGD dry eye phenotypes (Figures 2 and 3), indicating that WEs and some WdiEs in the meibum lipids are important for TFLL function. Of these, the loss of WEs may have a particularly large impact on the overall properties of meibum lipids since WEs are the most abundant meibum lipid class, together with CEs. The *Awat1* KO mice exhibited a weak MGD phenotype (Figures 2 and 3). We speculate that the reduced abundance of OAHFAs and type 1ω WdiEs in these mice partially impaired the formation of a lipid polarity gradient in the TFLL. We have previously reported that *Tg Cyp4f39* KO mice exhibit dry eye phenotypes (Miyamoto et al., 2020b). In those mice, the production of all ω-OH lipids (OAHFAs, type 1ω WdiEs, type 2ω WdiEs, and Chl-OAHFAs) was impaired (Figure 7D) (Miyamoto et al., 2020b). The dry eye phenotypes, in descending order of severity, in all four mouse lines are as follows: *Awat2* KO mice ≈ DKO mice > *Tg Cyp4f39* KO mice > *Awat1* KO mice. The fact that the dry eye phenotype of the *Tg Cyp4f39* KO mice was more severe than that of the *Awat1* KO mice may be the result of the additional reduction of type 2ω WdiEs and Chl-OAHFAs.

Recently, during the preparation of this manuscript, a paper investigating the dry eye phenotypes of *Awat2* KO mice was published (Widjaja-Adhi et al., 2020). Most of the dry eye phenotypes observed in that study are similar to those observed in the present study. In addition, in that study, consistent with our results (Figure 4), WE production was impaired in *Awat2* KO mice. However, there are some discrepancies between that paper and our results regarding the quantities of other meibum lipids. For example, that paper reported that WdiEs were not detected in WT mice but were in *Awat2* KO mice, which is inconsistent with our results (Figures 6 and 7). There are also differences in the quantities of CEs observed: in that study, the quantities of CEs were higher in *Awat2* KO mice than in WT mice, but in ours, they were equivalent (Figure 8C). The exact causes of the discrepancies are not clear at present but may have been caused by differences in sample preparation and measurement methods between the two studies: the method for obtaining and preparing the meibum lipids (squeezed from meibomian glands in the study by Widjaja-Adhi et al. versus whole meibomian glands in our study); the solvents used and number of extractions for lipid extraction (hexane once versus chloroform/methanol twice); the LC column (normal-phase column versus reverse-phase column); MS mode (single MS versus tandem MS [MS/MS]). Of these, we speculate that the differences in analytical systems are particularly significant. The lipid measurement in the study by Widjaja-Adhi et al. was conducted using LC-MS: lipids were separated by LC with a normal-phase column and by MS based on the *m/z* values of precursor ions. However, that method cannot be used to detect WdiEs with different types of FA/FAl chains separately. In meibum lipids, we know that type 1ω and type 2α/ω WdiEs are present, and other as yet unidentified lipid molecules that are structurally similar to WdiEs also seem to exist (Miyamoto et al., 2020b). Separation of these diverse lipids is therefore necessary. Furthermore, it is difficult to assign the LC peaks to particular lipid classes without confirmation using standards. Thus, it is highly likely that WdiEs (and possibly other lipids as well) were not accurately measured in that study (Widjaja-Adhi et al., 2020). Here, we have conducted lipid analyses using LC-MS/MS, where lipids are separated based on their differences in hydrophobicity (i.e., differences in chain length) by LC with a reversed-phase column and on the differences in the *m/z* values of both precursor and product ions. We also synthesized lipid classes by ourselves that are not commercially available (Figures 7 and 8) (Miyamoto et al., 2020b).

**Figure 9:**
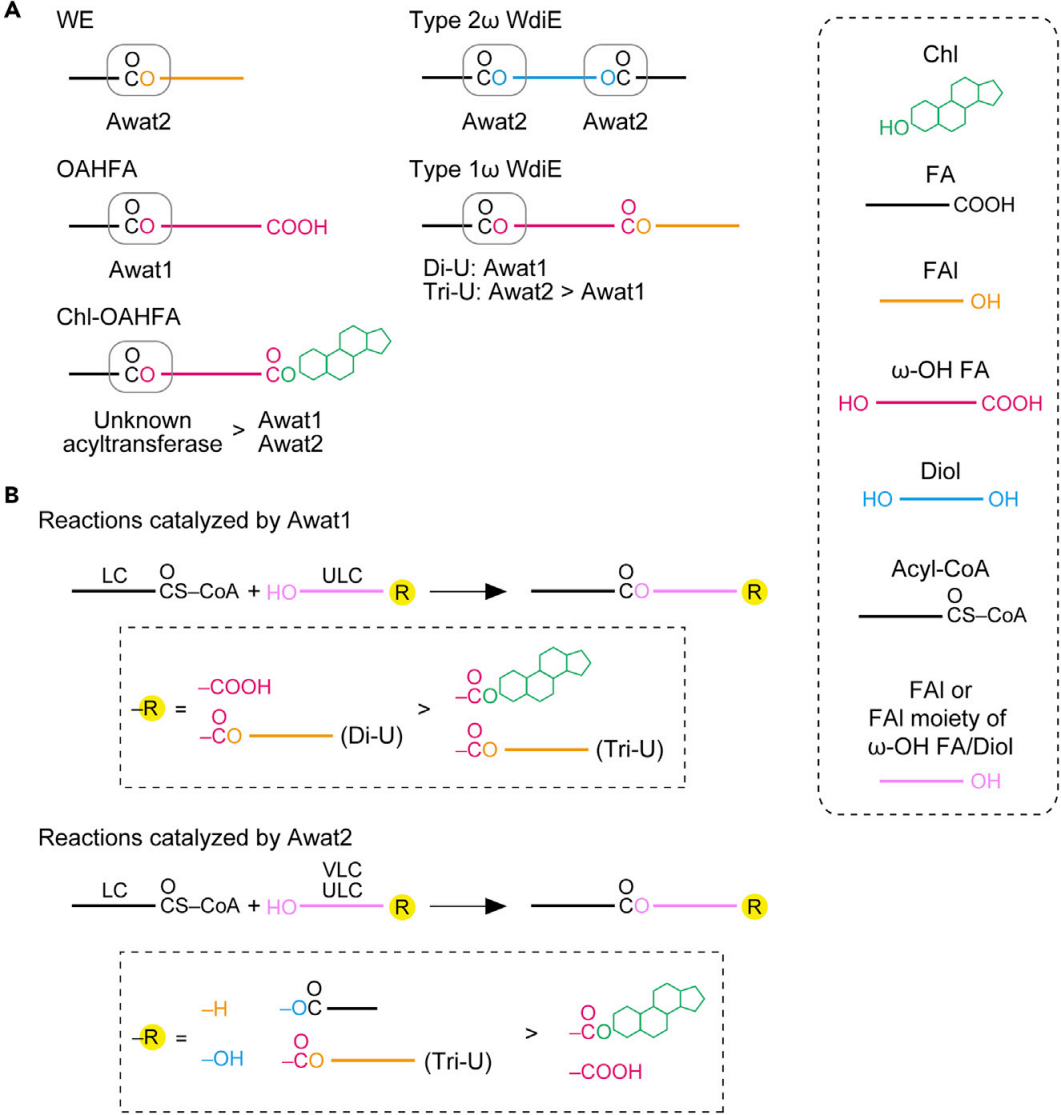
Model for the substrate specificity of Awat1 and Awat2 in the production of diverse meibum lipids (A) Models for the involvement of Awat1 and Awat2 in the formation of ester bonds (in gray boxes) to produce the indicated meibum lipids. Inequality signs denote the relative contribution of the enzymes involved. (B) Substrate specificity of Awat1 and Awat2 toward FAl and FAl derivatives. Di-U, di-unsaturated; Tri-U, tri-unsaturated.

Based on our present findings regarding changes in the meibum lipid composition in *Awat* KO/DKO mice, we propose the following model for the substrate specificity of Awat1 and Awat2. However, we cannot exclude other models, and our model will need to be validated by *in vitro* experiments in the future. The abundance of WEs was substantially reduced in the meibum lipids of *Awat2* KO mice (Figure 4). The WEs in mouse meibum lipids consist of a long-chain FA (mainly C16:1 or C18:1) and a C24-C30 VLC/ULCFAl (Figure 4 and Table S1). Therefore, Awat2 catalyzes the formation of an ester bond using a long-chain acyl-CoA and a VLC/ULCFAl as substrates (Figures 9A and 9B). Considering that there are few WEs containing VLC/ULCFAs in mouse meibum lipids (Figure 4 and Table S1), Awat2 has high substrate specificity toward long-chain acyl-CoAs, especially C16:1-CoA and C18:1-CoA.

In contrast, in the meibum lipids of *Awat1* KO mice, the abundance of OAHFAs was reduced (Figure 5). OAHFAs are composed of a long-chain FA and an ω-OH ULCFA. Thus, Awat1 catalyzes ester bond formation using a long-chain acyl-CoA and an ω-OH ULCFA as substrates (Figures 9A and 9B). As in WEs, the (non-hydroxy) FA moiety of OAHFAs is almost exclusively long chain (Miyamoto et al., 2020b), suggesting that like Awat2, Awat1 exhibits high substrate specificity toward long-chain acyl-CoAs. Since the contribution of Awat2 to OAHFA production is low, it is likely that it does not prefer FAls containing a carboxyl group at the opposite end to those with a free end as substrates (Figure 9B). Meibum OAHFAs contain mainly C16:1, C18:1, or C18:2 FAs (Miyamoto et al., 2020b). Of these, OAHFAs with a C16:1 FA seem to be derived from meibocytes since their levels were reduced in *Tg Cyp4f39* KO mice (Miyamoto et al., 2020b). It is possible that the others originate mainly from the duct epithelium of the meibomian glands. Quantities of OAHFAs with C16:0, C18:0, C18:1, or C18:2 FAs were higher in *Awat2* KO mice than in WT mice, and these species were also present in DKO mice in substantial quantities (Figure 5B), suggesting that the expression levels of unknown OAHFA-producing acyltransferases had increased in the meibocytes or duct epithelium to compensate for *Awat2* deficiency.

Awat2 is involved in the production of type 2ω WdiEs (Figure 6), which have a ULC diol in the middle and long-chain FAs at both ends. Type 2ω WdiEs thus have two ester bonds, and Awat2 may be involved in the formation of at least one of these and perhaps both (Figure 9A). This suggests that Awat2 can use FAls containing another hydroxyl group and/or a hydroxyl group + FA ester at the opposite end as substrates (Figure 9B). Awat1 is responsible for the production of di-unsaturated type 1ω WdiEs (Figure 7B), but both Awat1 and Awat2 are involved in the synthesis of tri-unsaturated type 1ω WdiEs, although the contribution of Awat2 is higher. Thus, the substrate specificity of Awat1 and Awat2 toward the ω-OH FA-FAl ester differs depending on the degree of unsaturation. Type 1ω WdiEs have a structure in which a long-chain FA, an ω-OH ULCFA, and a VLC/ULCFAl are connected by two ester bonds. Considering that the substrate specificity of Awat1 and Awat2 toward acyl-CoAs is high only for long-chain acyl-CoAs, Awat1/Awat2 may be involved in the formation of the ester bond between a long-chain FA and an ω-OH ULCFA but not in the formation of that between an ω-OH ULCFA and a VLC/ULCFAl (Figure 9A). An unknown acyltransferase is thus likely to be responsible for the formation of the latter ester bond.

Both Awat1 and Awat2 are only partly involved in the production of Chl-OAHFAs (Figure 8B), in which a long-chain FA, an ω-OH ULCFA, and a Chl are connected via two ester bonds. Again considering the substrate specificity of Awat1/Awat2 toward acyl-CoAs, they seem to be involved in the formation of the ester bond between a long-chain FA and an ω-OH ULCFA (Figure 9A). However, the presence of the Chl weakens their recognition of ω-OH ULCFA-Chl esters as substrates, and thus, an unknown acyltransferase may be primarily involved in the formation of this ester bond.

In summary, the substrate preference of Awat1 toward FAls is high for ω-OH ULCFAs and ω-OH ULCFA-VLC/ULCFAl esters (di-unsaturated) and low for ω-OH ULCFA-Chl esters and ω-OH ULCFA-VLC/ULCFAl esters (tri-unsaturated) (Figure 9B). In contrast, the substrate preference of Awat2 is high for VLC/ULCFAls, ULC diols, ULC diol-FA esters, and ω-OH ULCFA-VLC/ULCFAl esters (tri-unsaturated) and low for ω-OH ULCFAs and ω-OH ULCFA-Chl esters. However, the substrate preferences of Awat1/Awat2 may differ from this model, depending on the assumed reaction order. For example, in the production of type 1ω WdiEs, it is also possible that the ester bond between the FA and ω-OH FA is first created by Awat1/Awat2 and then another ester bond is produced by an unidentified acyltransferase.

AWAT1 and AWAT2 belong to the DGAT2 family. The mammalian DGAT2 family has seven members (DGAT2, AWAT1, AWAT2, DGAT2L6, and MOGAT1–3) (Liu et al., 2012; Turkish et al., 2005). Of these, DGAT2 and DGAT2L6 are diacylglycerol *O*-acyltransferases, which produce triacylglycerols using a diacylglycerol and a long-chain acyl-CoA as substrates. MOGAT1–3 are monoacylglycerol *O*-acyltransferases that produce diacylglycerols using a monoacylglycerol and a long-chain acyl-CoA as substrates. Thus, long-chain acyl-CoAs are the common substrates of the DGAT2 family, but the other substrate differs among members (AWATs: alcohol; DGATs: diacylglycerol; MOGATs: monoacylglycerol).

The presence of solidified meibum in the orifice of the meibomian glands of the *Awat2* KO and the DKO mice (Figure 2B) was probably the result of impaired production of WEs in the meibum lipids. The TFLL is primarily composed of CEs and WEs, which are the least polar lipids in mammals. They are characterized by long carbon chains (C24–C30) and branched ends (*iso*- or *anteiso*-) (Butovich, 2017; Nicolaides et al., 1981; Tanno et al., 2021). Their low polarity and characteristic structures may be necessary for them to fulfill their roles in the TFLL, i.e., preventing water evaporation from the aqueous layer, maintaining appropriate tear viscoelasticity, reducing the surface tension of the tear film, and promoting respreading of the tear film after blinks. Why are WEs required in addition to CEs in the meibum lipids? One of the reasons is probably their melting points. The melting point of CEs is much higher than that of WEs. For example, the melting point of the CE composed of a C18:1 FA and Chl is 46.5°C, whereas that of the WE composed of a C18:1 FA and a C18:1 FAl is −4°C (Iyengar and Schlenk, 1969; Mahadevan and Lundberg, 1962). Thus, CEs alone are solid at body temperature and cannot be secreted from the meibomian glands. However, a mixture of CEs and WEs has a melting point close to the surface temperature of the cornea (32°C) (Bron et al., 2004). Indeed, we observed appropriate melting point of the meibum lipids in WT mice (34°C), whereas large increases in *Awat2* KO (62°C) and DKO mice (57°C) (Figure 2D). It is likely that even the slight increase in melting point observed in *Awat1* KO mice (39°C) (Figure 2D) is responsible for the plugging in the meibomian gland orifice (Figure 2B).

### Limitations of the study

Here, we performed time-dependent analyses of dry eye phenotypes on *Awat2* KO mice using a relatively large number of samples (n = 10). The analyses we performed included measurements of BUT, corneal damage, and corneal irregularity (Figure 3). These detailed analyses should be extended to include *Awat1* KO and DKO mice in the future. We observed firm plugging of the meibomian gland orifice in *Awat2* KO and DKO mice (Figure 2). Although such plugging often leads to atrophy of the meibomian glands (Bron et al., 2017), we did not observe such atrophy, at least macroscopically, even in aged mice (Figure S2). Future microscopic and time-dependent analyses will be needed. In the present study, we have elucidated the detailed composition of several ester-bond-containing meibum lipids in mice and gained insights into their roles in dry eye prevention and the contributions of *Awat1* and *Awat2* to their production. Further studies are needed to reveal the structures of the as yet undetermined meibum lipid classes, determine the synthesis mechanism of each meibum lipid (e.g., the order of the synthesis reactions and the identity of the unknown acyltransferases), and examine the changes in meibum lipid composition in dry eye patients and the relationship of these changes to the pathology of dry eye disease.

## STAR★Methods

### Key resources table

**Table:** 

REAGENT or RESOURCE	SOURCE	IDENTIFIER
**Chemicals, peptides, and recombinant proteins**		

30% Acrylamide/bis solution, 19:1	BIO-RAD	Cat#1610154
Pentobarbital	Tokyo Chemical Industry	Cat#P0776; CAS: 57-33-0
Isoflurane	FUJIFILM Wako Pure Chemical	Cat#099-06571; CAS: 26675-46-7
Fluorescein	FUJIFILM Wako Pure Chemical	Cat#065-00252; CAS: 2321-07-5
Super Fix	Kurabo	Cat#KY-500
30-Hydroxy triacontanoic acid	Nagara Science	Cat#NS490102
Oleoyl chloride	FUJIFILM Wako Pure Chemical	Cat#329-79572; CAS: 112-77-6
Palmitoleyl alcohol	Merck	Cat#P1547; CAS: 10378-01-5
Cholesterol	Merck	Cat#C8667; CAS: 57-88-5
Behenyl oleate	Merck	Cat#O3255; CAS: 127566-70-5
C22:0 Cholesteryl ester	Avanti Polar Lipids	Cat#110875

**Critical commercial assays**		

BigDye Terminator v3.1 Cycle Sequencing Kit	Thermo Fisher Scientific	Cat#4337455
NucleoSpin RNA Kit	TAKARA Bio	Cat#U0955C
PrimeScript II 1st strand cDNA Synthesis Kit	TAKARA Bio	Cat#6210A
One Step TB Green PrimeScript RT-PCR Kit II	TAKARA Bio	Cat#RR086A
AMP+ MaxSpec Kit	Cayman Chemical	Cat#710000

**Experimental models: organisms/strains**		

Mouse: C57BL/6J	Japan SLC	N/A
Mouse: *Awat1*^*−/*Y^	This study	N/A
Mouse: *Awat2*^−/Y^	This study	N/A
Mouse: *Awat1*^−/Y^*Awat2*^−/Y^	This study	N/A
Mouse: *Tg Cyp4f39*^*−/−*^	Miyamoto et al., 2020b	N/A

**Oligonucleotides**		

Oligonucleotides for guide RNAs and primers for PCR, see Table S9	This study	

**Recombinant DNA**		

pX330-U6-Chimeric_BB-CBh-hSpCas9	Addgene	Cat#42230

**Software and algorithms**		

MassLynx software	Waters	N/A
Microsoft Excel software	Microsoft	N/A
JMP 13 software	SAS Institute	https://www.jmp.com/ja_jp/software/buy-jmp.html

**Other**		

CLEA Rodent Diet CE-2	CLEA Japan	N/A
Amersham Imager 600	Amersham Biosciences	N/A
Applied Biosystems 3130 Genetic Analyzer	Thermo Fisher Scientific	N/A
Stemi DV4 stereomicroscope	Carl Zeiss	N/A
Zone-Quick	AYUMI Pharmaceutical Corporation	N/A
VAPO SCAN AS-VT100RS closed-chamber evaporimeter	Asahi Biomed	N/A
SL-17 slit lamp	Kowa	N/A
RO8000 slit lamp	Luneau Technology Operations	N/A
LED Lumiloupe	Elaice	N/A
ATM-02 Melting Temperature Measurement Device	AS ONE Corporation	Cat#1-5804-02
Leica DM5000 B microscope	Leica Microsystems	N/A
DFC295 digital color camera	Leica Microsystems	N/A
HPTLC Silica gel 60 plates	Merck	Cat#105641
Xevo TQ-S LC-coupled triple quadrupole mass spectrometer	Waters	N/A

### Resource availability

#### Lead contact

Further information and requests for resources should be directed to and, where possible, will be fulfilled by the lead contact, Akio Kihara (kihara@pharm.hokudai.ac.jp).

#### Materials availability

All reagents used in this study will be made available on reasonable request to the lead contact.

#### Data and code availability

The published article includes all datasets generated or analyzed during this study. This study did not generate/analyze code.

### Experimental model and subject details

#### Mice

*Awat1* KO, *Awat2* KO, and *Awat1 Awat2* DKO mice were generated using the CRISPR/Cas9 system as follows. The guide RNAs for *Awat1* and *Awat2* were designed to target the 20 bases adjacent to the protospacer-adjacent motif sequences in exons 3 and 5, respectively. For each gene, a pair of oligonucleotides (*Awat1*, Awat1-F1/-R1; *Awat2*, Awat2-F1/-R1) (Table S9) containing the targeted sequences was annealed and cloned into the *Bbs*I site of the CRISPR/Cas9 vector pX330-U6-Chimeric_BB-CBh-hSpCas9 (Addgene, Watertown, MA, USA). Since the *Awat1* and *Awat2* genes are located within a 0.2 Mb region on the X chromosome, the creation of DKO mice by crossing *Awat1* and *Awat2* KO mice had been predicted to be difficult. Therefore, we co-injected the *Awat1*- and *Awat2*-targeting plasmids into fertilized eggs of C57BL/6J mice to disrupt either or both of these genes on the same chromosome. Genomic DNA was prepared from the tails of offspring and subjected to PCR to amplify the DNA fragments containing the target sequences for subsequent polyacrylamide gel electrophoresis and DNA sequencing. For the PCR, the following primer pairs were used: for polyacrylamide gel electrophoresis, Awat1-F2/-R2 and Awat2-F2/-R2; for DNA sequencing, Awat1-F3/-R3 and Awat2-F3/-R3 (Table S9).

Founder mice with a mutation in one or both of the genes were obtained, and they were crossed with C57BL/6J mice to establish the heterozygous *Awat1* KO, *Awat2* KO, and *Awat1 Awat2* DKO mouse lines. Heterozygous female *Awat1* KO, *Awat2* KO, and DKO mice were crossed with male C57BL/6J mice. Male mice thus generated were either WT or *Awat* KO mice (hemizygous), since both *Awat1* and *Awat2* are located on the X chromosome. We used male *Awat* KO mice (and their littermate WT male mice as controls) for our analyses. Most of the analyses were performed using 6-week-old mice. On the other hand, BUT and corneal damage scores were measured in 7- to 23-week-old mice, and eyelid observations were performed in 3-, 6-week-old, and 22- to 26-month-old mice.

Production of *Tg Cyp4f39* KO mice was described previously (Miyamoto et al., 2020b). Since whole-body *Cyp4f39* KO mice exhibit neonatal lethality due to skin barrier abnormalities (Miyamoto et al., 2020a), we used *Tg Cyp4f39* KO mice, in which *Cyp4f39* is expressed in the epidermis (and epithelium) under the control of the involucrin (*IVL*) promoter.

All the mice were housed under specific pathogen-free conditions at a room temperature of 22 ± 2°C and humidity of 55 ± 5%, with a 12 h light/12 h dark cycle and food (CLEA Rodent Diet CE-2, CLEA Japan, Tokyo, Japan) and water available *ad libitum*. All animal experiments were approved by the institutional animal care and use committee of Hokkaido University and the institutional review board of the Lion Corporation.

### Method details

#### Polyacrylamide gel electrophoresis

A 16% polyacrylamide gel (20 cm × 20 cm, 1 mm thick) was prepared by diluting 30% acrylamide/bis solution (19:1, Bio-Rad Laboratories, Hercules, CA, USA) to 16% in 1 × TBE buffer (130 mM Tris-HCl, 45 mM boric acid, 2.5 mM EDTA, pH 8.3). DNAs were run on the gel in 1 × TBE electrophoresis buffer for 3 h, stained with 0.5 μg/mL ethidium bromide, and detected using Amersham Imager 600 (Amersham Biosciences, Piscataway, NJ, USA).

#### DNA sequencing

Sanger sequencing was performed using a BigDye Terminator v3.1 Cycle Sequencing Kit (Thermo Fisher Scientific, Waltham, MA, USA) and an Applied Biosystems 3130 Genetic Analyzer (Thermo Fisher Scientific).

#### Real-time quantitative RT-PCR

Meibomian glands were prepared from the upper and lower eyelids of mice under a stereomicroscope (Stemi DV4; Carl Zeiss, Oberkochen, Germany) and subjected to total RNA preparation using a NucleoSpin RNA Kit (TAKARA Bio, Shiga, Japan). The total RNAs were converted to cDNAs using the PrimeScript II 1st strand cDNA Synthesis Kit (TAKARA Bio) and the oligo dT primer, and real-time quantitative RT-PCR was performed using 100 pg of cDNAs, primer pairs (Awat1-F4/-R4, Awat2-F4/-R4, Far1-F/-R, Far2-F/-R, Soat1-F/-R, Cyp4f39-F/-R, and Hprt-F/-R) (Table S9), and a One Step TB Green PrimeScript RT-PCR Kit II (TAKARA Bio).

#### Evaluation of dry eye phenotypes

We evaluated the eyeblink rate, tear quantity, water evaporation from the eye surface, BUT, corneal epithelial damage, and corneal surface irregularity of the mice. Mice were anesthetized via intraperitoneal injection of pentobarbital (0.05 mg/g body weight; Tokyo Chemical Industry, Tokyo, Japan) for tear quantity measurement or via the inhalation of isoflurane (FUJIFILM Wako Pure Chemical, Osaka, Japan) for the measurement of water evaporation, BUT, corneal epithelial damage, and corneal surface irregularity. For the measurement of eyeblink rate, the mice were held by hand, and the right side of their faces was recorded using a digital camera for 1 min. The number of eyeblinks was counted by visual examination of the video. Tear quantity was measured using the phenol red-thread test (Zone-Quick, AYUMI Pharmaceutical Corporation, Tokyo, Japan), according to the manufacturer’s instructions. Water evaporation from the eye surface was measured using a closed-chamber evaporimeter (AS-VT100RS, Asahi Biomed, Yokohama, Japan), as described previously (Sassa et al., 2018).

For BUT measurement, 1 μL of 0.5% (w/v) fluorescein (FUJIFILM Wako Pure Chemical) in saline was placed on the eyes of the mice, and they were manually forced to blink, to ensure that the fluorescein solution covered the entire eyeball surface. The eye surface was then observed using a slit lamp (SL-17, Kowa, Nagoya, Japan, or RO8000, Luneau Technology Operations, Pont-de-l’Arche, France) with a cobalt blue filter. The BUT is the elapsed time (in seconds) from the moment when the eye surface was uniformly stained with the fluorescein solution until the uniformity of the staining was lost.

We scored corneal epithelial damage after the BUT measurement as follows. Excess fluorescein solution was removed, and the eyes were briefly washed with saline. Corneal epithelial damage was then assessed using the slit lamp with a cobalt blue filter. We scored the corneal epithelial damage according to previous reports (Simsek et al., 2019).

We scored corneal surface irregularity as follows. A ring-shaped image from a white-light source (LED Lumiloupe, Elaice, Tokyo, Japan) was projected onto the corneal surface of an anesthetized mouse, and the corneal surface irregularity was scored between 0 and 5, according to a previous report (Choi et al., 2015). The grade was determined based on the extent of the distortion of the ring-shaped image: 0, no distortion; 1, distortion in one quarter of the ring; 2, distortion in two quarters; 3, distortion in three quarters; 4, distortion in all four-quarters; and 5, severe distortion, in which the image could not be recognized as ring-shaped.

#### Determination of melting points of meibum lipids

Meibomian glands were prepared from the upper eyelids of mice under a stereomicroscope, snap frozen in liquid nitrogen, and stored at −80°C until analysis. For analysis, the glands were removed from the freezer and subjected to pressure with forceps, causing them to extrude meibum from the orifices. A small piece of extruded meibum was placed on the hot plate of a Melting Temperature Measurement Device (ATM-02, AS ONE Corporation, Osaka, Japan) and heated from 20°C to 70°C at a rate of 1°C/min. The melting point of the meibum lipids was defined as the temperature at which they melted completely.

#### Hematoxylin and eosin staining

The eyelids were fixed with Super Fix (Kurabo, Osaka, Japan) at 4°C for ≥24 h. Preparation of paraffin sections and staining with hematoxylin and eosin were performed as described previously (Sassa et al., 2013). Brightfield images were captured under a Leica DM5000 B microscope equipped with a DFC295 digital color camera (Leica Microsystems, Wetzlar, Germany).

#### Chemical synthesis of type 1ω WdiE and Chl-OAHFA

For the synthesis of type 1ω WdiE and Chl-OAHFA, we first synthesized (*O*-C18:1)-ω-OH C30:0 FA essentially as described previously (Miyamoto et al., 2020b), with some modifications. 30-Hydroxy triacontanoic acid (ω-OH C30:0 FA; 1.0 mg, 2.1 μmol; Nagara Science, Gifu, Japan) was dissolved in 1 mL of tetrahydrofuran/hexane (1:1, v/v), mixed with triethylamine (1.3 μL, 9.2 μmol) and oleoyl chloride (2.3 μL, 6.9 μmol; FUJIFILM Wako Pure Chemical) on ice and incubated at room temperature for 48 h while being mixed. The octadec-10-enoic-30-(octadec-10-enoyloxy)-triacontanoic anhydride thus produced was hydrolyzed by incubating it with 20 μL of saturated aqueous sodium bicarbonate at room temperature for 6 h, producing sodium 30-(octadec-10-enoyloxy)-triacontanoate. Next, (*O*-C18:1)-ω-OH C30:0 FA was generated by adding 1 M hydrochloric acid to the above sodium salt until the pH of the sample was less than 3. Phase separation was performed by adding 300 μL of tetrahydrofuran/hexane (1:1, v/v) to the reaction solution, followed by centrifugation at 20,400 × *g* for 3 min at room temperature. The organic phase containing the (*O*-C18:1)-ω-OH C30:0 FA was recovered, dried, and dissolved in 500 μL of tetrahydrofuran.

A type 1ω WdiE, (*O*-C18:1)-ω-OH C30:0 FA–C16:1 FAl, was synthesized as follows (Figure S3). An aliquot (50 μL) of the (*O*-C18:1)-ω-OH C30:0 FA synthesized as described above was mixed with 350 μL of tetrahydrofuran, oxalyl chloride (10 μL, 0.12 mmol), and *N*,*N*-dimethylformamide (0.5 μL, 6.5 μmol) on ice, and this mixture was incubated at room temperature for 10 h while being mixed. The 30-(octadec-10-enoyloxy)-triacontanoic chloride thus produced was mixed with 4-dimethylaminopyridine (1 mg, 8.2 μmol), triethylamine (1 μL, 7.3 μmol), and palmitoleyl alcohol (20 μL, 70.6 μmol; Merck, Darmstadt, Germany), and incubated at room temperature overnight while being mixed, generating the type 1ω WdiE.

A Chl-OAHFA, (*O*-C18:1)–ω-OH C30:0 FA–Chl, was synthesized as follows (Figure S4). First, 30-(octadec-10-enoyloxy)-triacontanoic chloride was synthesized as described above from an aliquot (150 μL) of (*O*-C18:1)-ω-OH C30:0 FA mixed with 350 μL of tetrahydrofuran. After the reaction, 4-dimethylaminopyridine (1 mg, 8.2 μmol), triethylamine (1 μL, 7.3 μmol), and cholesterol (1.5 mg, 2.59 μmol; Merck) were added and incubated at room temperature overnight while being mixed, generating the Chl-OAHFA.

#### Lipid analyses by LC–MS/MS

Meibomian glands were prepared from the upper and lower eyelids of the mice under a stereomicroscope and subjected to lipid extraction as follows. Meibomian glands (2.4–15.6 mg) were subjected to lipid extraction in 600 μL of chloroform/methanol (1:2, v/v) in a glass homogenizer. The supernatant was recovered, and the remaining meibomian-gland tissue debris was subjected to a second extraction in another 600 μL of chloroform/methanol (1:2, v/v). The supernatant was recovered, combined with the previous one, and mixed with 400 μL of chloroform and 720 μL of water. Phase separation was performed by centrifugation (9,000 × *g*, room temperature, 3 min), and the organic phase (total lipid fraction) was recovered and dried. The total lipids corresponding to 0.1 mg of meibomian gland tissues were dissolved in 200 μL of hexane. The lipids were then subjected to a second phase separation via mixing with 200 μL of water and centrifugation (20,400 × *g*, room temperature, 3 min). The organic phase was recovered, and the lipids were re-extracted from the aqueous phase via mixing with 200 μL of hexane, followed by centrifugation. The combined organic phase (meibum lipid fraction) was dried. The OAHFAs in the meibum lipid fraction were derivatized with *N*-(4-aminomethylphenyl)pyridinium (AMPP) using an AMP+ MaxSpec Kit (Cayman Chemical, Ann Arbor, MI, USA) as described previously (Hancock et al., 2018; Miyamoto et al., 2020b).

WEs and CEs were purified from the total lipids as follows to reduce the background noise in the MS analysis. The total lipids corresponding to 1 mg of meibomian gland tissues were suspended in chloroform/methanol (1:2, v/v) and resolved by thin-layer chromatography (TLC) on Silica Gel 60 HPTLC plates (Merck), using hexane/toluene (1:1, v/v) as the solvent system. The region of silica gel containing WEs/CEs was scraped collectively from the TLC plate and incubated with 300 μL of ethanol at 37°C for 1 h. The ethanol fraction was recovered by centrifugation (20,400 × *g*, room temperature, 3 min), and the residual silica gel was rinsed with 300 μL of ethanol. After centrifugation as above, this rinsed fraction was combined with the previous ethanol fraction and dried. The lipids were dissolved in 200 μL of hexane, then mixed with 200 μL of 10% methanol and subjected to phase separation by centrifugation (20,400 × *g*, room temperature, 3 min). The hexane phase, which contained the WEs and CEs, was recovered and dried.

LC–MS/MS analyses were performed using ultra-performance LC (UPLC) coupled with electrospray ionization tandem triple-quadrupole mass spectrometer (Xevo TQ-S; Waters, Milford, MA, USA), as described previously (Miyamoto et al., 2020b). The lipids corresponding to 5 ng of meibomian gland tissues were injected onto the UPLC column. Lipid separation by UPLC was performed at a flow rate of 0.3 mL/min using a gradient system in which mobile phase A (acetonitrile/water [3:2, v/v] containing 5 mM ammonium formate) and mobile phase B (acetonitrile/2-propanol [1:9, v/v] containing 5 mM ammonium formate) were mixed. The gradient conditions were as follows: 0 min, 60% B; 0–21 min, gradient to 100% B; 21–25 min, 100% B; 25–30 min, 60% B. Ionization was performed via electrospray ionization in the positive ion mode, with the cone voltage set to 35 V. Quantitative analyses were performed in MRM mode, using the following collision energies for each lipid class: WEs and WdiEs, 20 eV; OAHFA-AMPPs, 60 eV; Chl-OAHFAs and CEs, 15 eV. The *m*/*z* values of the precursor and product ions of each lipid species were set in the mass filters Q1 and Q3, respectively (Tables S10, S11, S12, S13, S14, and S15). Behenyl oleate (C18:1 FA–C22:0 FAl, Merck) and C22:0 CE (Avanti Polar Lipids, Alabaster, AL, USA) were used as external standards for the quantification of WEs and CEs, respectively. Fragment ion analyses of the chemically synthesized type 1ω WdiE, (*O*-C18:1)-ω-OH C30:0 FA–C16:1 FAl, and the synthesized Chl-OAHFA, (*O*-C18:1)-ω-OH C30:0 FA–Chl, were performed by product-ion scanning using the following settings: precursor ion, *m*/*z* = 955.9 for the type 1ω WdiE and *m*/*z* = 1102.1 for the Chl-OAHFA; collision energy, 15 eV for both lipids. Data analyses were performed using MassLynx software (Waters).

### Quantification and statistical analysis

Eyeblink rate, tear quantity, and water evaporation were measured using the right eye only, due to technical problems, but all other assays were performed on both eyes. There were no differences in the measurement results between left and right eyes. Data are presented as mean ± SD. The significance of differences between groups was evaluated using Student’s *t-*test in Microsoft Excel (Microsoft, Redmond, WA, USA) or Tukey’s test, the Tukey–Kramer test, or Dunnett’s test in JMP 13 (SAS Institute, Cary, NC, USA). A p-value of <0.05 was considered significant.

## Supplemental information

Document S1. Figures S1–S4 and Tables S1, S2, S7–S11, S14, and S15

Table S3. Quantities of all type 2ω WdiEs measured in this study, Related to Figures 6A and 6B

Table S4. Quantities of all type 2α WdiEs measured in this study, Related to Figures 6C and 6D

Table S5. Quantities of all type 1ω WdiEs measured in *Awat1* KO, *Awat2* KO, and DKO mice, Related to Figures 7B and 7C

Table S6. Quantities of all type 1ω WdiEs measured in *Tg Cyp4f39* KO mice, Related to Figure 7D

Table S12. Selected *m/z* values for type 2ω/α WdiEs in MS/MS analysis, Related to Figures 6A–6D

Table S13. Selected *m/z* values for type 1ω WdiEs in MS/MS analysis, Related to Figures 7B–7D

Document S2. Article plus supplemental information
