# Diverse meibum lipids produced by Awat1 and Awat2 are important for stabilizing tear film and protecting the ocular surface

**DOI:** 10.1016/j.isci.2021.103160

**Published:** 2021-09-25

**Authors:** Megumi Sawai, Keisuke Watanabe, Kana Tanaka, Wataru Kinoshita, Kento Otsuka, Masatoshi Miyamoto, Takayuki Sassa, Akio Kihara

## Main text

(iScience *24*, 102478-1–102478-17.e18–e22; May 21, 2021)

In the originally published article, the values in Table S12 were duplicated in Table S13. In the current version of the Table S13, we have listed the correct values. The correction of this error does not affect any of the reported results or conclusions of the paper but nevertheless should be stated correctly. The authors sincerely apologize to the readers for this unintended lack of thoroughness and any confusion that may have resulted from that.

