# Supplementary material for: Diverse meibum lipids produced by Awat1 and Awat2 are important for stabilizing tear film and protecting the ocular surface
Source: iScience. 2021 Apr 26;24(5):102478. doi: 10.1016/j.isci.2021.102478 (PMC8169949; doi:10.1016/j.isci.2021.102478)
Supplement: Document S1. Figures S1–S4 and Tables S1, S2, S7–S11, S14, and S15 [file mmc1.pdf]

iScience, Volume *24*

Supplemental information

Diverse meibum lipids produced by Awat1 and Awat2 are important for stabilizing tear film and protecting the ocular surface

Megumi Sawai, Keisuke Watanabe, Kana Tanaka, Wataru Kinoshita, Kento Otsuka, Masatoshi Miyamoto, Takayuki Sassa, and Akio Kihara

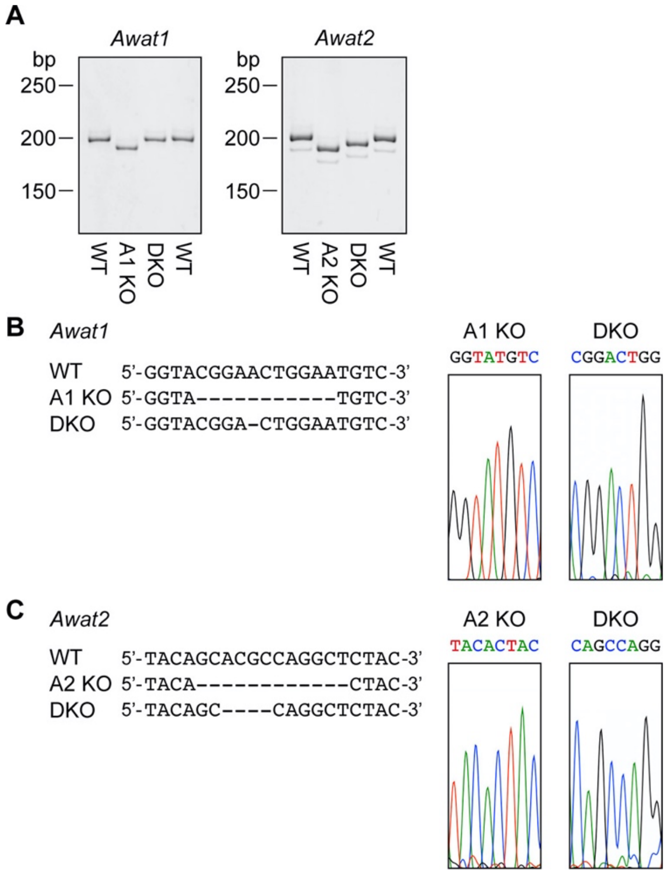

Figure S1. Gene Disruption of *Awat1* and/or *Awat2* in KO/DKO Mice. Related to Figures 1B and 1C.

(A) DNA fragments around the guide RNA target sequences in *Awat1* and *Awat2* were amplified by PCR using tail DNA from WT, *Awat1* KO, *Awat2* KO, and *Awat1 Awat2* DKO mice, separated by 16% polyacrylamide gel electrophoresis, and stained with ethidium bromide. bp, base pairs.

(B and C) Mutations in *Awat1* in *Awat1* KO and DKO mice (B) and *Awat2* in *Awat2* KO and DKO mice (C), determined by DNA sequencing. Sanger sequencing chromatograms of four bases each on the 5′ and 3′ ends of the deletion are shown.

A1 KO, *Awat1* KO; A2 KO, *Awat2* KO.

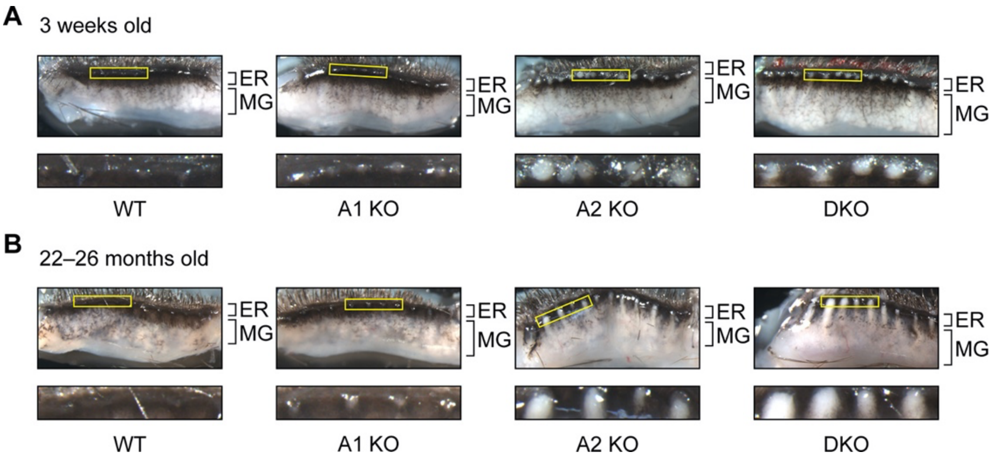

Figure S2. Meibomian Glands and Their Orifices in Young and Old Mice. Related to Figure 2B.

Upper eyelids from WT, *Awat1* KO, *Awat2* KO, and *Awat1 Awat2* DKO mice at 3 weeks old (A) and 22 or 26 months old (WT and *Awat1* KO mice, 22 months old; *Awat2* KO and DKO mice, 26 months old) (B), photographed under a light microscope. The lower images are magnifications of the yellow rectangles in the upper images and show the meibomian gland (MG) orifices.

A1 KO, *Awat1* KO; A2 KO, *Awat2* KO; ER, eyelid rim.

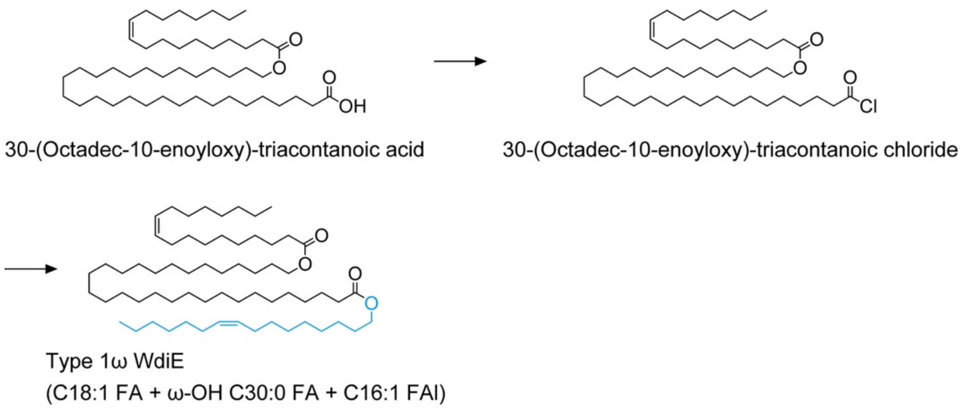

Figure S3. Synthesis scheme for type 1ω WdiE, (*O*-C18:1)-ω-OH C30:0–C16:1. Related to Figure 7.

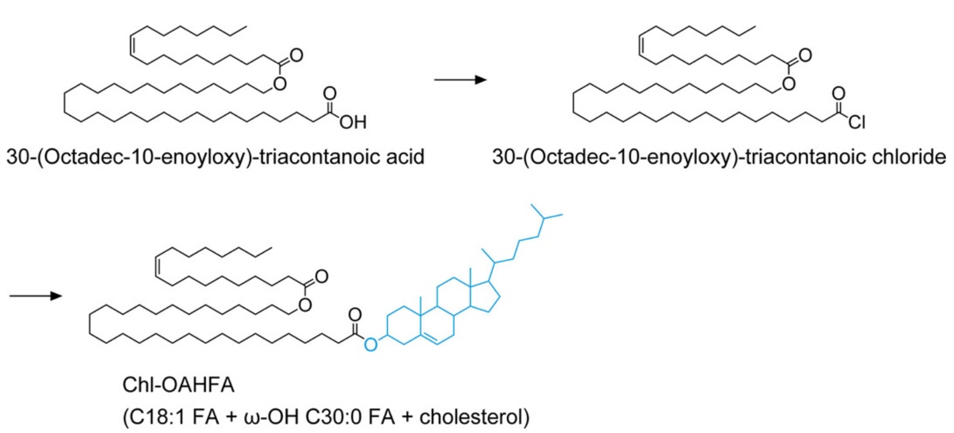

Figure S4. Synthesis scheme for Chl-OAHFA, (*O*-C18:1)–ω-OH C30:0–Chl. Related to Figure 8.

Table S1. Quantities of all WEs measured in this study. Related to Figures 4A and 4B.

FA	FAl	WT (pmol/mg)	A1 KO (pmol/mg)	A2 KO (pmol/mg)	DKO (pmol/mg)
C16:1	C16:1	ND	ND	ND	ND
C16:1	C17:1	ND	ND	ND	ND
C16:1	C18:1	ND	ND	ND	ND
C16:1	C19:1	ND	ND	ND	ND
C16:1	C20:1	0.09 ± 0.03	0.11 ± 0.06	0.13 ± 0.10	0.11 ± 0.11
C16:1	C21:1	ND	ND	ND	ND
C16:1	C22:1	ND	ND	ND	ND
C16:1	C23:1	ND	ND	ND	ND
C16:1	C24:1	0.42 ± 0.28	0.52 ± 0.45	0.13 ± 0.12	0.13 ± 0.10
C16:1	C25:1	ND	ND	ND	ND
C16:1	C26:1	6.67 ± 3.94	6.25 ± 6.36	0.10 ± 0.12	0.03 ± 0.02
C16:1	C27:1	0.42 ± 0.17	0.41 ± 0.31	0.00 ± 0.00	0.00 ± 0.00
C16:1	C28:1	8.37 ± 3.74	6.56 ± 6.08	0.04 ± 0.09	0.01 ± 0.02
C16:1	C29:1	0.63 ± 0.27	0.45 ± 0.46	0.00 ± 0.00	0.00 ± 0.00
C16:1	C30:1	26.71 ± 13.19	19.89 ± 18.23	0.04 ± 0.07	0.00 ± 0.01
C16:1	C31:1	1.82 ± 0.87	0.66 ± 0.51	0.00 ± 0.00	0.00 ± 0.00
C16:1	C32:1	54.18 ± 27.87	32.70 ± 33.74	0.20 ± 0.25	0.07 ± 0.08
C16:1	C33:1	2.70 ± 1.63	1.84 ± 2.14	0.00 ± 0.01	0.00 ± 0.00
C16:1	C34:1	7.63 ± 5.05	4.34 ± 5.58	0.02 ± 0.02	0.03 ± 0.03
C16:1	C35:1	0.28 ± 0.28	0.25 ± 0.29	0.03 ± 0.04	0.00 ± 0.00
C16:1	C36:1	0.08 ± 0.09	0.02 ± 0.04	0.00 ± 0.00	0.00 ± 0.00
C16:1	C16:0	0.02 ± 0.03	0.03 ± 0.01	0.07 ± 0.12	0.03 ± 0.04
C16:1	C17:0	ND	ND	ND	ND
C16:1	C18:0	0.07 ± 0.07	0.12 ± 0.10	0.16 ± 0.10	0.14 ± 0.12
C16:1	C19:0	ND	ND	ND	ND
C16:1	C20:0	0.17 ± 0.16	0.17 ± 0.16	0.25 ± 0.14	0.16 ± 0.07
C16:1	C21:0	0.18 ± 0.23	0.18 ± 0.19	0.02 ± 0.03	0.02 ± 0.03
C16:1	C22:0	1.51 ± 2.74	0.36 ± 0.35	0.18 ± 0.26	0.06 ± 0.06
C16:1	C23:0	1.42 ± 2.07	0.60 ± 0.55	0.17 ± 0.25	0.06 ± 0.04
C16:1	C24:0	24.01 ± 12.26	21.29 ± 14.47	0.46 ± 0.56	0.30 ± 0.18
C16:1	C25:0	32.58 ± 14.77	34.34 ± 25.50	0.17 ± 0.21	0.07 ± 0.04

C16:1	C26:0	348.86 ± 144.37	261.96 ± 222.25	1.53 ± 1.79	0.31 ± 0.24
C16:1	C27:0	146.57 ± 47.58	100.79 ± 70.35	0.43 ± 0.68	0.14 ± 0.11
C16:1	C28:0	43.95 ± 17.31	26.26 ± 20.48	0.44 ± 0.44	0.18 ± 0.16
C16:1	C29:0	19.89 ± 7.01	11.42 ± 7.95	0.10 ± 0.09	0.02 ± 0.02
C16:1	C30:0	10.73 ± 5.19	6.50 ± 6.44	0.14 ± 0.11	0.06 ± 0.05
C16:1	C31:0	6.85 ± 3.82	3.69 ± 3.58	0.04 ± 0.04	0.04 ± 0.04
C16:1	C32:0	1.77 ± 1.43	1.12 ± 1.29	0.04 ± 0.05	0.04 ± 0.04
C16:1	C33:0	0.64 ± 0.39	0.47 ± 0.57	0.00 ± 0.01	0.01 ± 0.01
C16:1	C34:0	ND	ND	ND	ND
C16:1	C35:0	ND	ND	ND	ND
C16:1	C36:0	ND	ND	ND	ND
C16:2	C26:0	0.96 ± 0.49	0.88 ± 0.67	0.10 ± 0.02	0.07 ± 0.04
C18:2	C26:0	7.15 ± 2.75	4.32 ± 4.09	0.09 ± 0.06	0.07 ± 0.03
C16:1	C26:0	330.65 ± 121.26	234.02 ± 212.13	1.70 ± 1.58	0.73 ± 0.47
C17:1	C26:0	7.75 ± 3.29	4.89 ± 4.23	0.07 ± 0.08	0.02 ± 0.00
C18:1	C26:0	149.93 ± 52.51	80.66 ± 64.86	1.89 ± 1.47	1.18 ± 0.80
C19:1	C26:0	0.45 ± 0.17	0.21 ± 0.17	0.03 ± 0.01	0.02 ± 0.00
C20:1	C26:0	0.63 ± 0.44	0.31 ± 0.26	0.06 ± 0.02	0.09 ± 0.06
C21:1	C26:0	ND	ND	ND	ND
C22:1	C26:0	0.14 ± 0.10	0.07 ± 0.07	0.05 ± 0.03	0.11 ± 0.08
C23:1	C26:0	ND	ND	ND	ND
C24:1	C26:0	0.08 ± 0.04	0.07 ± 0.05	0.18 ± 0.10	0.36 ± 0.20
C25:1	C26:0	ND	ND	ND	ND
C26:1	C26:0	ND	ND	ND	ND
C16:0	C26:0	ND	ND	ND	ND
C17:0	C26:0	ND	ND	ND	ND
C18:0	C26:0	ND	ND	ND	ND
C19:0	C26:0	ND	ND	ND	ND
C20:0	C26:0	ND	ND	ND	ND
C21:0	C26:0	0.28 ± 0.03	0.16 ± 0.08	0.10 ± 0.03	0.07 ± 0.04
C22:0	C26:0	ND	ND	ND	ND
C23:0	C26:0	0.11 ± 0.02	0.07 ± 0.04	0.07 ± 0.02	0.06 ± 0.02
C24:0	C26:0	ND	ND	ND	ND
C25:0	C26:0	ND	ND	ND	ND

C26:0	C26:0	ND	ND	ND	ND

Values represent the means ± standard deviations (n = 5). ND, not detected.

Table S2. Quantities of all OAHFAs measured in this study. Related to Figures 5A and 5B.

FA	ω-OH FA	WT (×10^5 peak area/mg)	A1 KO (×10^5 peak area/mg)	A2 KO (×10^5 peak area/mg)	DKO (×10^5 peak area/mg)
C16:1	C16:1	ND	ND	ND	ND
C16:1	C17:1	ND	ND	ND	ND
C16:1	C18:1	ND	ND	ND	ND
C16:1	C19:1	ND	ND	ND	ND
C16:1	C20:1	ND	ND	ND	ND
C16:1	C21:1	ND	ND	ND	ND
C16:1	C22:1	ND	ND	ND	ND
C16:1	C23:1	ND	ND	ND	ND
C16:1	C24:1	0.14 ± 0.05	0.40 ± 0.12	0.01 ± 0.01	0.00 ± 0.00
C16:1	C25:1	ND	ND	ND	ND
C16:1	C26:1	0.12 ± 0.02	0.17 ± 0.12	0.05 ± 0.04	0.05 ± 0.05
C16:1	C27:1	ND	ND	ND	ND
C16:1	C28:1	ND	ND	ND	ND
C16:1	C29:1	ND	ND	ND	ND
C16:1	C30:1	0.67 ± 0.21	0.12 ± 0.10	0.44 ± 0.14	0.13 ± 0.11
C16:1	C31:1	0.39 ± 0.29	0.04 ± 0.04	0.08 ± 0.10	0.07 ± 0.02
C16:1	C32:1	13.62 ± 2.20	2.03 ± 0.39	9.96 ± 2.08	2.03 ± 0.57
C16:1	C33:1	7.70 ± 0.67	2.24 ± 0.62	3.32 ± 0.84	0.69 ± 0.18
C16:1	C34:1	24.24 ± 2.38	4.96 ± 1.23	19.23 ± 4.75	5.76 ± 2.27
C16:1	C35:1	14.66 ± 1.40	7.32 ± 1.90	4.09 ± 0.80	1.19 ± 0.26
C16:1	C36:1	4.04 ± 0.23	0.02 ± 0.04	0.00 ± 0.00	0.00 ± 0.00
C16:1	C16:0	ND	ND	ND	ND
C16:1	C17:0	ND	ND	ND	ND
C16:1	C18:0	ND	ND	ND	ND
C16:1	C19:0	ND	ND	ND	ND
C16:1	C20:0	ND	ND	ND	ND
C16:1	C21:0	ND	ND	ND	ND
C16:1	C22:0	0.10 ± 0.04	0.21 ± 0.12	0.03 ± 0.02	0.01 ± 0.01
C16:1	C23:0	0.18 ± 0.06	0.25 ± 0.12	0.03 ± 0.06	0.01 ± 0.02
C16:1	C24:0	0.36 ± 0.13	0.55 ± 0.12	0.18 ± 0.05	0.21 ± 0.07
C16:1	C25:0	ND	ND	ND	ND

C16:1	C26:0	0.27 ± 0.14	0.25 ± 0.07	0.44 ± 0.11	0.78 ± 0.28
C16:1	C27:0	0.20 ± 0.04	0.07 ± 0.06	0.02 ± 0.01	0.02 ± 0.01
C16:1	C28:0	0.01 ± 0.01	0.01 ± 0.01	0.08 ± 0.10	0.20 ± 0.18
C16:1	C29:0	ND	ND	ND	ND
C16:1	C30:0	0.32 ± 0.05	0.16 ± 0.07	0.23 ± 0.09	0.34 ± 0.16
C16:1	C31:0	0.39 ± 0.07	0.02 ± 0.02	0.09 ± 0.07	0.09 ± 0.05
C16:1	C32:0	0.39 ± 0.12	0.14 ± 0.11	0.33 ± 0.15	0.34 ± 0.19
C16:1	C33:0	1.35 ± 0.26	0.34 ± 0.18	0.26 ± 0.05	0.17 ± 0.08
C16:1	C34:0	ND	ND	ND	ND
C16:1	C35:0	0.21 ± 0.07	0.09 ± 0.07	0.00 ± 0.00	0.02 ± 0.02
C16:1	C36:0	ND	ND	ND	ND
C16:2	C34:1	0.59 ± 0.36	0.03 ± 0.04	0.46 ± 0.32	0.03 ± 0.04
C18:2	C34:1	6.72 ± 3.60	3.93 ± 1.20	35.41 ± 16.75	35.63 ± 6.10
C16:1	C34:1	18.88 ± 5.78	2.95 ± 1.32	20.58 ± 9.28	7.02 ± 2.10
C17:1	C34:1	0.91 ± 0.79	0.10 ± 0.07	1.27 ± 0.88	0.37 ± 0.20
C18:1	C34:1	11.16 ± 2.87	2.67 ± 0.38	47.81 ± 20.55	26.12 ± 6.67
C19:1	C34:1	ND	ND	ND	ND
C20:1	C34:1	0.08 ± 0.05	0.03 ± 0.02	1.27 ± 0.91	0.91 ± 0.40
C21:1	C34:1	ND	ND	ND	ND
C22:1	C34:1	0.09 ± 0.03	0.01 ± 0.01	0.67 ± 0.32	0.33 ± 0.23
C23:1	C34:1	ND	ND	ND	ND
C24:1	C34:1	ND	ND	ND	ND
C25:1	C34:1	ND	ND	ND	ND
C26:1	C34:1	0.03 ± 0.02	0.01 ± 0.01	0.35 ± 0.35	0.08 ± 0.11
C27:1	C34:1	ND	ND	ND	ND
C28:1	C34:1	ND	ND	ND	ND
C29:1	C34:1	ND	ND	ND	ND
C30:1	C34:1	ND	ND	ND	ND
C31:1	C34:1	ND	ND	ND	ND
C32:1	C34:1	ND	ND	ND	ND
C33:1	C34:1	ND	ND	ND	ND
C34:1	C34:1	ND	ND	ND	ND
C35:1	C34:1	ND	ND	ND	ND
C36:1	C34:1	ND	ND	ND	ND
C16:0	C34:1	2.17 ± 0.96	0.81 ± 0.47	11.95 ± 5.07	5.14 ± 1.27

C17:0	C34:1	1.22 ± 0.36	0.08 ± 0.07	0.64 ± 0.14	0.36 ± 0.09
C18:0	C34:1	0.10 ± 0.11	0.15 ± 0.05	5.89 ± 3.04	2.15 ± 1.29
C19:0	C34:1	0.02 ± 0.01	ND	0.29 ± 0.27	0.08 ± 0.03
C20:0	C34:1	0.01 ± 0.01	0.01 ± 0.00	0.34 ± 0.21	0.08 ± 0.06
C21:0	C34:1	ND	ND	ND	ND
C22:0	C34:1	ND	ND	ND	ND
C23:0	C34:1	ND	ND	ND	ND
C24:0	C34:1	ND	ND	ND	ND
C25:0	C34:1	ND	ND	ND	ND
C26:0	C34:1	ND	ND	ND	ND
C27:0	C34:1	ND	ND	ND	ND
C28:0	C34:1	ND	ND	ND	ND
C29:0	C34:1	ND	ND	ND	ND
C30:0	C34:1	ND	ND	ND	ND
C31:0	C34:1	ND	ND	ND	ND
C32:0	C34:1	ND	ND	ND	ND
C33:0	C34:1	ND	ND	ND	ND
C34:0	C34:1	ND	ND	ND	ND
C35:0	C34:1	ND	ND	ND	ND
C36:0	C34:1	ND	ND	ND	ND

Values represent the means ± standard deviations (n = 4). ND, not detected.

Table S7. Quantities of all Chl-OAHFAs measured in this study. Related to Figure 8B.

OAHFA	WT (×10^5 peak area/mg)	A1 KO (×10^5 peak area/mg)	A2 KO (×10^5 peak area/mg)	DKO (×10^5 peak area/mg)
C32:3	ND	ND	ND	ND
C33:3	ND	ND	ND	ND
C34:3	ND	ND	ND	ND
C35:3	ND	ND	ND	ND
C36:3	ND	ND	ND	ND
C37:3	ND	ND	ND	ND
C38:3	ND	ND	ND	ND
C39:3	ND	ND	ND	ND
C40:3	ND	ND	ND	ND
C41:3	ND	ND	ND	ND
C42:3	ND	ND	ND	ND
C43:3	ND	ND	ND	ND
C44:3	ND	ND	ND	ND
C45:3	ND	ND	ND	ND
C46:3	ND	ND	ND	ND
C47:3	ND	ND	ND	ND
C48:3	0.92 ± 0.33	0.10 ± 0.12	0.04 ± 0.06	0.05 ± 0.07
C49:3	0.25 ± 0.07	0.07 ± 0.06	0.02 ± 0.01	0.02 ± 0.02
C50:3	2.10 ± 0.55	1.01 ± 0.11	0.65 ± 0.47	0.33 ± 0.38
C51:3	0.22 ± 0.08	0.12 ± 0.04	0.03 ± 0.03	0.04 ± 0.01
C52:3	1.43 ± 0.29	0.92 ± 0.13	0.75 ± 0.30	0.51 ± 0.13
C53:3	ND	ND	ND	ND
C54:3	0.54 ± 0.14	0.33 ± 0.22	0.48 ± 0.15	0.39 ± 0.06
C32:2	ND	ND	ND	ND
C33:2	0.00 ± 0.00	0.00 ± 0.00	0.05 ± 0.05	0.26 ± 0.08
C34:2	ND	ND	ND	ND
C35:2	0.00 ± 0.00	0.00 ± 0.00	0.08 ± 0.09	0.24 ± 0.16
C36:2	ND	ND	ND	ND
C37:2	0.00 ± 0.00	0.00 ± 0.00	0.08 ± 0.07	0.17 ± 0.06
C38:2	ND	ND	ND	ND
C39:2	ND	ND	ND	ND

C40:2	ND	ND	ND	ND
C41:2	ND	ND	ND	ND
C42:2	ND	ND	ND	ND
C43:2	ND	ND	ND	ND
C44:2	ND	ND	ND	ND
C45:2	ND	ND	ND	ND
C46:2	0.54 ± 0.19	0.25 ± 0.09	0.14 ± 0.17	0.43 ± 0.19
C47:2	0.35 ± 0.19	0.04 ± 0.05	0.02 ± 0.01	0.04 ± 0.04
C48:2	5.54 ± 1.59	2.69 ± 0.26	1.85 ± 0.57	2.61 ± 0.22
C49:2	1.21 ± 0.40	0.59 ± 0.24	0.22 ± 0.04	0.60 ± 0.13
C50:2	10.53 ± 2.70	8.05 ± 0.41	5.45 ± 1.26	7.35 ± 1.21
C51:2	0.95 ± 0.33	0.84 ± 0.25	0.49 ± 0.16	0.83 ± 0.10
C52:2	6.13 ± 1.34	5.24 ± 1.53	3.54 ± 0.95	5.23 ± 0.83
C53:2	0.35 ± 0.13	0.29 ± 0.05	0.13 ± 0.04	0.29 ± 0.13
C54:2	3.24 ± 1.14	1.89 ± 0.88	1.98 ± 0.52	4.17 ± 0.44

Values represent the means ± standard deviations (n = 4). ND, not detected.

Table S8. Quantities of all CEs measured in this study. Related to Figure 8C.

FA	WT (pmol/mg)	A1 KO (pmol/mg)	A2 KO (pmol/mg)	DKO (pmol/mg)
C16:1	22.18 ± 32.88	22.27 ± 34.49	4.07 ± 5.69	1.14 ± 2.54
C17:1	0.85 ± 1.91	1.12 ± 2.50	0.89 ± 1.98	0.46 ± 1.02
C18:1	23.31± 17.68	16.51 ± 21.67	ND	2.17 ± 3.02
C19:1	ND	0.64 ± 1.43	ND	ND
C20:1	21.52± 15.05	8.07 ± 11.41	ND	1.74 ± 3.88
C21:1	3.71 ± 5.14	0.80 ± 1.79	0.64 ± 1.44	ND
C22:1	65.41± 22.55	55.12 ± 75.31	13.77 ± 13.99	6.63 ± 9.76
C23:1	ND	ND	ND	ND
C24:1	69.01± 24.86	67.33 ± 91.29	35.93 ± 21.57	40.41 ± 22.28
C25:1	ND	ND	1.65 ± 2.60	0.81 ± 1.81
C26:1	29.48± 20.23	25.45 ± 42.12	19.55 ± 17.15	47.85 ± 48.83
C27:1	ND	0.70 ± 1.58	0.91 ± 2.03	4.82 ± 8.58
C28:1	26.04 ± 27.98	27.26 ± 41.00	43.61 ± 22.48	49.36 ± 39.70
C29:1	1.21 ± 2.70	ND	0.82 ± 1.83	1.91 ± 2.75
C30:1	45.56 ± 40.67	24.33 ± 29.95	41.08 ± 16.82	87.86 ± 76.86
C31:1	ND	0.44 ± 0.99	1.90 ± 4.26	5.46 ± 10.35
C32:1	34.94 ± 50.99	19.74 ± 31.57	37.32 ± 28.30	78.49 ± 51.68
C33:1	6.24 ± 10.20	2.26 ± 3.37	3.01 ± 4.66	7.14 ± 7.11
C34:1	12.23 ± 15.49	0.82 ± 1.84	10.89 ± 7.55	15.45 ± 15.14
C35:1	0.69 ± 1.54	0.70 ± 1.57	0.89 ± 1.22	2.20 ± 2.26
C36:1	ND	0.92 ± 2.05	ND	ND
C16:0	127.67 ± 40.32	56.93 ± 72.17	75.48 ± 32.08	39.48 ± 32.54
C17:0	97.57 ± 23.59	48.53 ± 34.68	29.48 ± 27.62	21.80 ± 8.49
C18:0	60.36 ± 35.87	19.06 ± 38.81	12.69 ± 6.62	36.12 ± 24.53
C19:0	106.41 ± 20.52	54.38 ± 46.04	50.73 ± 22.57	36.13 ± 35.33
C20:0	456.30 ± 178.01	234.26 ± 152.02	296.93 ± 119.79	250.95 ± 89.87
C21:0	400.19 ± 234.69	213.39 ± 131.97	264.36 ±147.67	209.08 ± 72.41
C22:0	291.23 ± 258.19	141.82 ± 112.36	170.76 ± 112.92	114.98 ± 69.25
C23:0	208.05 ± 122.08	102.86 ± 109.70	140.15 ± 87.56	93.53 ± 27.23
C24:0	309.64 ± 176.15	166.68 ± 176.88	194.46 ± 62.38	171.31 ± 82.26
C25:0	465.79 ± 230.73	344.12 ± 375.26	274.93 ± 88.59	262.12 ± 117.22
C26:0	447.40 ± 290.43	262.46 ± 219.09	285.13 ± 102.06	294.55 ± 124.56

C27:0	252.27 ± 138.95	165.83 ± 153.67	163.98 ± 61.53	162.95 ± 86.84
C28:0	47.96 ± 52.54	30.56 ± 39.70	34.95 ± 41.68	39.81 ± 27.95
C29:0	42.21 ± 38.81	25.34 ± 26.43	20.34 ± 11.32	23.04 ± 27.12
C30:0	4.61 ± 6.56	4.58 ± 10.25	6.49 ± 8.24	1.82 ± 3.51
C31:0	5.36 ± 5.21	3.11 ± 5.49	3.74 ± 3.83	3.56 ± 3.47
C32:0	1.26 ± 2.09	ND	0.55 ± 1.22	ND
C33:0	ND	ND	0.42 ± 0.93	ND
C34:0	ND	ND	ND	ND
C35:0	ND	0.48 ± 1.08	1.68 ± 2.31	2.40 ± 5.36
C36:0	ND	ND	ND	0.27 ± 0.60

Values represent the means ± standard deviations (n = 5). ND, not detected.

Table S9. List of oligonucleotides used. Related to Figures 1B–E.

Oligonucleotide name	Sequence
Awat1-F1	5'-CACCGGTTCAGACTGGGTACGGAAC-3'
Awat1-R1	5'-AAACGTTCCGTACCCAGTCTGAACC-3'
Awat1-F2	5'-GGGTGAATAAGAGACCTGGGGTGG-3'
Awat1-R2	5'-TCCCTAGTCATTTGATATAATGTCTGC-3'
Awat1-F3	5'-GGGATCCAAACGCTAGACAGCTGAAACCATTC-3'
Awat1-R3	5'-CACATTGTAGCTACTTCTCTGAAGC-3'
Awat1-F4	5'-GGAGAAACAGAGGTATATGACCAGG-3'
Awat1-R4	5'-TCACAAGAATATCAGCTTCTGGGTGTTGG-3'
Awat2-F1	5'-CACCGGAGTGCAGATACAGCACGCC-3'
Awat2-R1	5'-AAACGGCGTGCTGTATCTGCACTCC-3'
Awat2-F2	5'-GGCTTTTTCTGCACACTAGGGTCGTG-3'
Awat2-R2	5'-CCTTACCCATGTTTAAGGGCTGTGC-3'
Awat2-F3	5'-GGGATCCCAAGATTGTCTCAGGTGCTAACCGC-3'
Awat2-R3	5'-AGTAAATACACACACACATGCATGC-3'
Awat2-F4	5'-GGAGAGACAGACCTCTATGACCAGC-3'
Awat2-R4	5'-TCAAACTATCACCAGCTCCTGGGTC-3'
Far1-F	5'-GATAATGTCAATATGTTAATGAACC-3'
Far1-R	5'-TCAGTATCTCATAGTGCTGGATGCTCG-3'
Far2-F	5'-TCCATGCTGGAGTATTTCATCAACC-3'
Far2-R	5'-TTGAACAAGGGACAAATGAAGAACC-3'
Soat1-F	5'-GCCGTCTTCGCCCTGTCGGCTGTGG-3'
Soat1-R	5'-CTAAAACACGTACCGACAAGTCCAGG -3'
Cyp4f39-F	5'-AGCATCTACGGGACCCACCACAACC-3'
Cyp4f39-R	5'-TGAGGGTAGAGGCTCTACATTGAGC-3'
Hprt-F	5'-GCTGACCTGCTGGATTACATTAAAG-3'
Hprt-R	5'-CTTAACCATTTTGGGGCTGTACTGC-3'

Table S10. Selected *m/z* values for WEs in MS/MS analysis. Related to Figures 4A and 4B.

FA	FAl	Precursor ion (Q1) $[M + H]^+$	Product ion (Q3) $[FA - OH]^+$
C16:1	C16:1	477.5	237.1
C16:1	C17:1	491.5	237.1
C16:1	C18:1	505.5	237.1
C16:1	C19:1	519.5	237.1
C16:1	C20:1	533.5	237.1
C16:1	C21:1	547.5	237.1
C16:1	C22:1	561.6	237.1
C16:1	C23:1	575.6	237.1
C16:1	C24:1	589.6	237.1
C16:1	C25:1	603.6	237.1
C16:1	C26:1	617.6	237.1
C16:1	C27:1	631.6	237.1
C16:1	C28:1	645.6	237.1
C16:1	C29:1	659.7	237.1
C16:1	C30:1	673.7	237.1
C16:1	C31:1	687.7	237.1
C16:1	C32:1	701.7	237.1
C16:1	C33:1	715.7	237.1
C16:1	C34:1	729.7	237.1
C16:1	C35:1	743.8	237.1
C16:1	C36:1	757.8	237.1
C16:1	C16:0	479.5	237.1
C16:1	C17:0	493.5	237.1
C16:1	C18:0	507.5	237.1
C16:1	C19:0	521.5	237.1
C16:1	C20:0	535.5	237.1
C16:1	C21:0	549.6	237.1
C16:1	C22:0	563.6	237.1
C16:1	C23:0	577.6	237.1
C16:1	C24:0	591.6	237.1
C16:1	C25:0	605.6	237.1

C16:1	C26:0	619.6	237.1
C16:1	C27:0	633.6	237.1
C16:1	C28:0	647.7	237.1
C16:1	C29:0	661.7	237.1
C16:1	C30:0	675.7	237.1
C16:1	C31:0	689.7	237.1
C16:1	C32:0	703.7	237.1
C16:1	C33:0	717.7	237.1
C16:1	C34:0	731.8	237.1
C16:1	C35:0	745.8	237.1
C16:1	C36:0	759.8	237.1
C16:2	C26:0	617.6	235.1
C18:2	C26:0	645.6	263.1
C17:1	C26:0	633.6	251.1
C18:1	C26:0	647.7	265.1
C19:1	C26:0	661.7	279.1
C20:1	C26:0	675.7	293.1
C21:1	C26:0	689.7	307.1
C22:1	C26:0	703.7	321.2
C23:1	C26:0	717.7	335.2
C24:1	C26:0	731.8	349.2
C25:1	C26:0	745.8	363.2
C26:1	C26:0	759.8	377.2
C16:0	C26:0	621.6	239.1
C17:0	C26:0	635.7	253.1
C18:0	C26:0	649.7	267.1
C19:0	C26:0	663.7	281.1
C20:0	C26:0	677.7	295.1
C21:0	C26:0	691.7	309.2
C22:0	C26:0	705.7	323.2
C23:0	C26:0	719.8	337.2
C24:0	C26:0	733.8	351.2
C25:0	C26:0	747.8	365.2
C26:0	C26:0	761.8	379.2

Table S11. Selected *m/z* values for OAHFAs in MS/MS analysis. Related to Figures 5A and 5B.

FA	ω-OH FA	Precursor ion (Q1) [M + AMPP]$^{+}$	Product ion (Q3) [M + AMPP − (FA − OH)]$^{+}$
C16:1	C16:1	673.5	419.0
C16:1	C17:1	687.5	433.0
C16:1	C18:1	701.5	447.0
C16:1	C19:1	715.5	461.0
C16:1	C20:1	729.5	475.1
C16:1	C21:1	743.5	489.1
C16:1	C22:1	757.6	503.1
C16:1	C23:1	771.6	517.1
C16:1	C24:1	785.6	531.1
C16:1	C25:1	799.6	545.1
C16:1	C26:1	813.6	559.1
C16:1	C27:1	827.6	573.2
C16:1	C28:1	841.6	587.2
C16:1	C29:1	855.7	601.2
C16:1	C30:1	869.7	615.2
C16:1	C31:1	883.7	629.2
C16:1	C32:1	897.7	643.2
C16:1	C33:1	911.7	657.3
C16:1	C34:1	925.7	671.3
C16:1	C35:1	939.8	685.3
C16:1	C36:1	953.8	699.3
C16:1	C16:0	675.5	421.0
C16:1	C17:0	689.5	435.0
C16:1	C18:0	703.5	449.0
C16:1	C19:0	717.5	463.1
C16:1	C20:0	731.5	477.1
C16:1	C21:0	745.6	491.1
C16:1	C22:0	759.6	505.1
C16:1	C23:0	773.6	519.1
C16:1	C24:0	787.6	533.1
C16:1	C25:0	801.6	547.1

C16:1	C26:0	815.6	561.2
C16:1	C27:0	829.6	575.2
C16:1	C28:0	843.7	589.2
C16:1	C29:0	857.7	603.2
C16:1	C30:0	871.7	617.2
C16:1	C31:0	885.7	631.2
C16:1	C32:0	899.7	645.3
C16:1	C33:0	913.7	659.3
C16:1	C34:0	927.8	673.3
C16:1	C35:0	941.8	687.3
C16:1	C36:0	955.8	701.3
C16:2	C34:1	923.7	671.3
C18:2	C34:1	951.7	671.3
C17:1	C34:1	939.7	671.3
C18:1	C34:1	953.7	671.3
C19:1	C34:1	967.7	671.3
C20:1	C34:1	981.8	671.3
C21:1	C34:1	995.8	671.3
C22:1	C34:1	1009.8	671.3
C23:1	C34:1	1023.8	671.3
C24:1	C34:1	1037.8	671.3
C25:1	C34:1	1051.8	671.3
C26:1	C34:1	1065.9	671.3
C27:1	C34:1	1079.9	671.3
C28:1	C34:1	1093.9	671.3
C29:1	C34:1	1107.9	671.3
C30:1	C34:1	1121.9	671.3
C31:1	C34:1	1135.9	671.3
C32:1	C34:1	1150.0	671.3
C33:1	C34:1	1164.0	671.3
C34:1	C34:1	1178.0	671.3
C35:1	C34:1	1192.0	671.3
C36:1	C34:1	1206.0	671.3
C16:0	C34:1	927.7	671.3
C17:0	C34:1	941.7	671.3

C18:0	C34:1	955.7	671.3
C19:0	C34:1	969.8	671.3
C20:0	C34:1	983.8	671.3
C21:0	C34:1	997.8	671.3
C22:0	C34:1	1011.8	671.3
C23:0	C34:1	1025.8	671.3
C24:0	C34:1	1039.8	671.3
C25:0	C34:1	1053.9	671.3
C26:0	C34:1	1067.9	671.3
C27:0	C34:1	1081.9	671.3
C28:0	C34:1	1095.9	671.3
C29:0	C34:1	1109.9	671.3
C30:0	C34:1	1123.9	671.3
C31:0	C34:1	1138.0	671.3
C32:0	C34:1	1152.0	671.3
C33:0	C34:1	1166.0	671.3
C34:0	C34:1	1180.0	671.3
C35:0	C34:1	1194.0	671.3
C36:0	C34:1	1208.0	671.3

Table S14. Selected *m/z* values for Chl-OAHFAs in MS/MS analysis. Related to Figure 8B.

OAHFA	Precursor ion (Q1) $[M + H]^+$	Product ion (Q3) $[Chl - H_2O]^+$
C32:3	873.8	369.4
C33:3	887.8	369.4
C34:3	901.9	369.4
C35:3	915.9	369.4
C36:3	929.9	369.4
C37:3	943.9	369.4
C38:3	957.9	369.4
C39:3	971.9	369.4
C40:3	986.0	369.4
C41:3	1000.0	369.4
C42:3	1014.0	369.4
C43:3	1028.0	369.4
C44:3	1042.0	369.4
C45:3	1056.0	369.4
C46:3	1070.1	369.4
C47:3	1084.1	369.4
C48:3	1098.1	369.4
C49:3	1112.1	369.4
C50:3	1126.1	369.4
C51:3	1140.1	369.4
C52:3	1154.1	369.4
C53:3	1168.2	369.4
C54:3	1182.2	369.4
C32:2	875.8	369.4
C33:2	889.9	369.4
C34:2	903.9	369.4
C35:2	917.9	369.4
C36:2	931.9	369.4
C37:2	945.9	369.4
C38:2	959.9	369.4
C39:2	974.0	369.4

C40:2	988.0	369.4
C41:2	1002.0	369.4
C42:2	1016.0	369.4
C43:2	1030.0	369.4
C44:2	1044.0	369.4
C45:2	1058.1	369.4
C46:2	1072.1	369.4
C47:2	1086.1	369.4
C48:2	1100.1	369.4
C49:2	1114.1	369.4
C50:2	1128.1	369.4
C51:2	1142.1	369.4
C52:2	1156.2	369.4
C53:2	1170.2	369.4
C54:2	1184.2	369.4

Table S15. Selected *m/z* values for CEs in MS/MS analysis. Related to Figure 8C.

FA	Precursor ion (Q1) $[M + H]^+$	Product ion (Q3) $[Chl – H_2O]^+$
C16:1	623.6	369.4
C17:1	637.6	369.4
C18:1	651.6	369.4
C19:1	665.6	369.4
C20:1	679.7	369.4
C21:1	693.7	369.4
C22:1	707.7	369.4
C23:1	721.7	369.4
C24:1	735.7	369.4
C25:1	749.7	369.4
C26:1	763.7	369.4
C27:1	777.8	369.4
C28:1	791.8	369.4
C29:1	805.8	369.4
C30:1	819.8	369.4
C31:1	833.8	369.4
C32:1	847.8	369.4
C33:1	861.9	369.4
C34:1	875.9	369.4
C35:1	889.9	369.4
C36:1	903.9	369.4
C16:0	625.6	369.4
C17:0	639.6	369.4
C18:0	653.6	369.4
C19:0	667.7	369.4
C20:0	681.7	369.4
C21:0	695.7	369.4
C22:0	709.7	369.4
C23:0	723.7	369.4
C24:0	737.7	369.4
C25:0	751.7	369.4

C26:0	765.8	369.4
C27:0	779.8	369.4
C28:0	793.8	369.4
C29:0	807.8	369.4
C30:0	821.8	369.4
C31:0	835.8	369.4
C32:0	849.9	369.4
C33:0	863.9	369.4
C34:0	877.9	369.4
C35:0	891.9	369.4
C36:0	905.9	369.4
